# The Design, Synthesis and Evaluation of Rho-kinase Inhibitory Activity of 4-aryl-thiazole-2-amines

**DOI:** 10.22037/ijpr.2020.114468.14866

**Published:** 2021

**Authors:** Linan Wang, Ben Ouyang, Meixia Fan, Junhui Qi, Lei Yao

**Affiliations:** *School of Pharmacy, Key Laboratory of Molecular Pharmacology and Drug Evaluation (Yantai University), Ministry of Education, Collaborative Innovation Center of Advanced Drug Delivery System and Biotech Drugs in Universities of Shandong, Yantai University, Yantai 264003, China.*

**Keywords:** 4-aryl-thiazole-2-amine, Inhibitors, Kinase, ROCK, Synthesis

## Abstract

Rho-associated kinases (ROCK) are a class of serine/threonine kinases that play important roles in various biological processes. ROCK are becoming attractive targets for drug designing. A novel scaffold was designed according to molecular hybridization strategy, then a series of 4-aryl-5-aminomethyl-thiazole-2-amines were synthesized, and their inhibitory activities on ROCK were screened by enzyme-linked immunosorbent assay (ELISA). The results showed that 4-aryl-5-aminomethyl-thiazole-2-amines derivatives displayed certain ROCK II inhibitory activities. The IC_50_ value of the most potent compound 4v was found to be 20 nM. The preliminary structure-activity-relationship investigation showed that compounds with 4-pyridine substitution were generally found to be more potent than compounds with 3-pyridine substitution. The molecular docking studies indicated that more optimization work needs to conduct to obtain more potent ROCK inhibitors.

## Introduction

**Figure 1:**
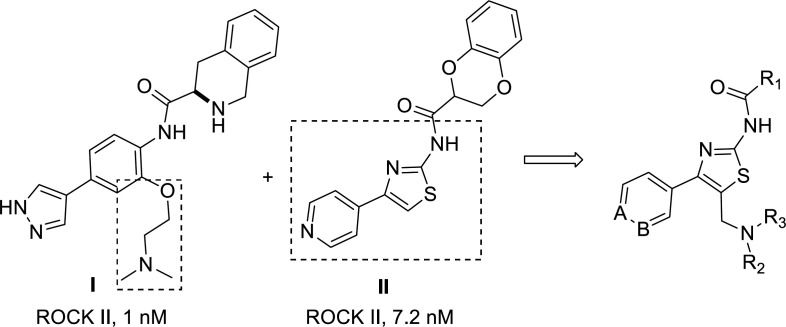
Structures of Fasudil and ROCK inhibitors under clinical trials

Rho-associated kinases (ROCK) are a class of serine/threonine kinases that play important roles in various biological processes such as smooth muscle contraction, cell apoptosis, cell migration, and proliferation (1-3). ROCK is becoming an attractive target for drug designing, and ROCK inhibitors are believed to have potential in the treatment of diseases such as hypertension (4, 5), glaucoma (6-9), cancer (10-12), and spinal cord injury (13). Currently, Fasudil and Ripasudil have been approved for clinical use in the treatment of cardiovascular and ophthalmic diseases (14). Meanwhile, as shown in Figure 1, several ROCK inhibitors are under pre-clinical investigational studies and clinical trials (15-17). 

**Figure 2:**
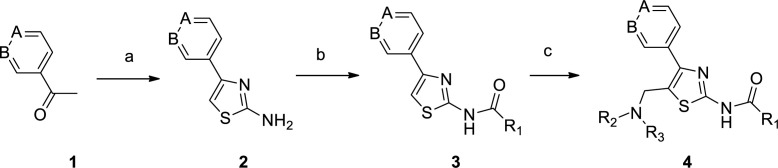
The design of 4-aryl-5-aminoalkyl-thiazole-2-amines

ROCK contains two isoforms, ROCK I and ROCK II identified that share approximately 92% sequence identity within the kinase domain (18, 19). Due to the difference in distribution, ROCK I and ROCK II are responsible for different physiological functions. ROCK II is widely distributed in the brain and heart, so selective ROCK II inhibitors have been attracting more attention in recent years (1, 20 and 21). Previously, we identified that tetrahydroisoquinoline derivatives, such as compound** I** in Figure 2, as potent and selective ROCK II inhibitors (22, 23). The structure-activity-relationship and molecular docking studies showed that introducing the side aminoalkyl chain could enhance the ROCK isomer selectivity. However, undesired pharmacokinetic properties, such as high clearance and low bioavailability, were not suitable for non-topical administration of compound **I**. Compound **II **(Figure 2), a pyridine-thiazole-based amide, showed potent *in-vitro* enzyme-based ROCK II inhibitory activity (24). However, it was much less potent in a cell-based assay (25). In order to find novel ROCK inhibitors suitable for systematic administration from these leads, 4-aryl-5-aminoalkyl-thiazole-2-amines (Figure 2) were designed according to the molecular hybridization strategy. These structures incorporated both the aminomethyl side chain and thiazolamine moieties of compounds **I **and **II**. 

## Experimental

*Chemistry*

*General*

Reagents of the highest commercial quality were purchased and used without further purification unless otherwise specified. 

*Synthesis of 4-(Pyridin-4-yl) thiazol-2-amine (2)*

Step 1. To a solution of 4-acetylpyridine (4.90 g, 41.30 mmol) and 48% HBr (7.0 mL) in acetic acid (46.0 mL), a solution of bromine (2.30 mL, 45.00 mmol) in acetic acid (8.0 mL) at 0 °C was added dropwise. After addition, the reaction mixture was allowed to stir at 70 °C for 1 h. The mixture was cooled to 0 °C and treated with diethyl ether. The resultant white solid was isolated by vacuum filtration to give 9.90 g (87%) of bromoketone as the HBr salt, which was used in the next step as obtained. 

Step 2. A mixture of the above intermediate (10.00 g, 35.59 mmol), thiourea (2.71 g, 35.59 mmol), and absolute EtOH (100 mL) was refluxed overnight. After cooling to room temperature, the reaction mixture was diluted with water (400 mL), and the pH was adjusted to 11 with concentrated ammonium hydroxide solution and was further stirred for 2 h. The resulting precipitate was filtered, washed, and dried to provide the title compound as a yellowish solid (5.85 g, 93% yield). (m.p. 270-272 ºC) (26). 

*Synthesis of 4-Methyl-N-(4-(pyridine-4-yl) thiazol-2-yl) benzamide (3)*

To a solution of *p-*toluic acid (1.10 g, 5.64 mmol) in 10 mL DCM, EDCI (1.60 g, 8.46 mmol) and HOBt (0.76 g, 5.64 mmol) were added at room temperature. The mixture was stirred at room temperature for 30 min, then a solution of compound** 2 (**1.00 g, 5.64 mmol) in 10 mL DCM was added slowly. The resulting reaction mixture was stirred at room temperature for 8 h until TLC indicated that all the starting material was consumed. The reaction mixture was diluted with water and extracted with DCM. The organic phase was washed with water and brine, dried, filtered, and concentrated. The residue was purified by chromatography to afford the title compound **3 **(1.62 g, 83% yield) as a yellow solid and used in the next step.

*Synthesis of 5-aryl-4-aminomethyl-thiazole -2-amines (4)*

To a mixture of compound** 3** (1.9 g, 10.7 mmol, 1.0 equiv.), appropriate amine (1.2 equiv.) in acetic acid was paraformaldehyde (3 equiv.) at room temperature. The reaction mixture was allowed to stir at 70 °C for 1 h. After removing the extra acetic acid under reduced pressure, the residue was diluted with ethyl acetate. The solution was neutralized with saturated sodium bicarbonate. The organic layer was separated, washed with water and brine, dried, filtered, and concentrated. The residue was purified by flash chromatography to afford the title compound.

*4-Methyl-N-(5-(morpholinomethyl)-4-(pyridin-3-yl) thiazol-2-yl) benzamide (4a)*

White solid. m.p.198-199 °C; ^1^H NMR (400 MHz, CD_3_OD) δ: 9.29-9.30 (m, 1H, ArH), 8.94-8.98 (m, 2H, ArH), 8.21-8.25 (m, 1H, ArH), 7.91-7.93 (m, 2H, ArH), 7.37-7.39 (m, 2H, ArH), 4.86-4.92 (m, 2H, ArCH_2_), 3.87-3.99 (m, 4H, 2XOCH_2_) 3.50 (s, 2H, CH_2_), 3.23 (s, 2H, CH2), 2.43 (s, 3H, CH_3_); ^13^C NMR (125 MHz, DMSO-*d*6) δ: 164.8, 159.3, 145.6, 143.1, 142.9, 142.8, 142.3, 131.8, 128.7, 128.0, 127.7, 125.9, 116.1, 65.7, 62.5, 49.8, 49.5, 30.1, 20.5; HRMS calcd. for C_21_H_23_N_4_O_2_S [M^+^+1] 395.1536, found 395.1533.

*4-Methyl-N-(5-((4-methylpiperazin-1-yl) methyl)-4-(pyridin-3-yl) thiazol-2-yl) benz-amide (4b)*

White solid. m.p. 180-182 ℃; ^1^H NMR (400 MHz, CD_3_OD) δ: 9.37 (s, 1H, ArH), 9.06 (s, 1H, ArH), 8.88 (s, 1H, ArH), 8.22 (s, 1H, ArH), 7.93 (s, 2H, ArH), 7.38 (s, 2H, ArH), 4.36 (s, 2H, ArCH_2_), 3.30-3.65 (m, 4H, 2XNCH_2_), 2.95 (s, 3H, CH_3_), 2.44 (s, 3H, CH_3_); ^13^C NMR (125 MHz, DMSO-*d*6) δ: 165.8, 158.9, 144.5, 143.8, 142.5, 141.8, 133.5, 129.7, 129.2, 128.8, 127.5, 66.8, 50.9, 48.4, 42.3, 21.6; HRMS calcd. for C_22_H_26_N_5_OS [M^+^+1] 408.1853, found 408.1847.

*N-(5-((i-Propyl(methyl)amino) methyl)-4-(pyridin-3-yl) thiazol-2-yl)-4-methylbenzamide (4c)*

 White solid. m.p. 184-185 ℃; ^1^H NMR (400 MHz, MeOD) δ: 8.90-8.91(m, 1H, ArH), 8.84-8.85 (m, 1H, ArH), 8.16-8.19 (m, 1H, ArH), 7.91-7.93 (m, 2H, ArH), 7.50-7.53 (m, 1H, ArH), 7.35-7.37 (m, 2H, ArH), 3.84 (s, 2H, CH_2_), 2.94-3.01 (m, 1H, CH), 2.43 (s, 3H, CH_3_), 2,23 (s, 3H, CH_3_), 1.05 (t, *J *= 6.8 Hz, 6H, 2CH_3_); ^13^C NMR (125 MHz, DMSO-*d*6) δ: 165.4, 157.7, 149.5, 148.8, 143.3, 142.3, 136.0, 131.3, 130.3, 129.7, 129.6, 128.7, 123.9, 53.0, 49.8, 36.9, 21.6, 18.0; HRMS calcd. for C_21_H_25_N_4_OS [M^+^+1] 381.1744, found 381.1749. 

*3,4-Dichloro-N-(5-(morpholinomethyl)-4-(pyridin-3-yl) thiazol-2-yl) benzamide (4d)*

White solid. m.p. 208-210 ℃; ^1^H NMR (400 MHz, DMSO-*d*6) δ: 8.84-8.85 (m, 1H, ArH), 8.53-8.55 (m, 1H, ArH), 8.33-8.34 (m, 1H, ArH), 8.00-8.04 (m, 2H, ArH), 7.78-7.80 (m, 1H, ArH), 7.45-7.47 (m, 1H, ArH), 3.73 (s, 2H, CH_2_), 3.54-3.56 (m, 4H, 2XOCH_2_), 2.42 (m, 4H, 2XNCH_2_); ^13^C NMR (100 MHz, DMSO-*d*6) δ: 167.5, 151.7, 149.6, 149.1, 136.2, 136.0, 133.1, 132.3, 132.1, 131.5, 130.7, 129.3, 129.0, 124.8, 124.1, 66.8, 54.4, 53.7; HRMS calcd. for C_20_H_19_Cl_2_N_4_O_2_S [M^+^+1] 449.0600, found 449.0604. 

*3,4-Dichloro-N-(5-((4-methylpiperazin-1-yl) methyl)-4-(pyridin-3-yl) thiazol-2-yl) benzamide (4e)*

White solid. m.p. 179-181 ℃; ^1^H NMR (400 MHz, DMSO-*d*6) δ: 8.84-8.85 (m, 1H, ArH), 8.53-8.55(m, 1H, ArH), 8.34-8.35 (m, 1H, ArH), 8.02-8.03 (m, 2H, ArH), 8.00-8.01(m, 1H, ArH), 7.79-7.81(m, 1H, ArH), 3.74 (s, 2H, CH_2_), 2.46-2.47 (m, 8H, 4XNCH_2_), 2.24 (s, 3H, CH_3_); ^13^C NMR (100 MHz, DMSO-*d*6) δ: 163.7,149.6, 149.1, 144.4, 143.3, 136.2, 136.0, 133.0, 132.1, 131.5, 131.0, 130.7, 128.9,127.7,124.1, 54.7, 53.8, 52.3, 45.3; HRMS calcd. for C_ 21_H_22_Cl_2_N_5_OS [M^+^+1] 462.0917, found 462.0911.

*3,4-Dichloro-N-(5-((i-propyl(methyl)amino) methyl)-4-(pyridin-3-yl) thiazol-2-yl) benzamide (4f)*

Yellow solid. m.p. 171-172 ℃; ^1^H NMR (400 MHz, DMSO-*d*6) δ: 12.82 (br s, 1H, NH), 8.87-8.88 (m, 1H, ArH), 8.56-8.57 (m, 1H, ArH), 8.37-8.38 (m, 1H, ArH), 8.05-8.06 (m, 2H, ArH), 7.83-7.85 (m, 1H, ArH), 7.49-7.51(m, 1H, ArH), 3.80 (s, 2H, ArCH_2_), 2.91-2.92 (m, 1H, CH), 2.16 (s, 3H, CH_3_), 1.10 (s, 6H, 2CH_3_); ^13^C NMR (100 MHz, DMSO-*d*6) δ: 163.6, 151.3, 149.5, 149.0, 136.0, 135.9, 133.2, 132.0, 131.5, 131.2, 130.9, 130.7,128.9, 124.0, 123.0, 53.2, 49.9, 37.0, 18.1; HRMS calcd. for C_20_H_21_Cl_2_N_4_OS [M^+^+1] 435.0808, found 435.0811.

*N-(5-((4-Methylpiperazin-1-yl) methyl)-4-(pyridin-3-yl) thiazol-2-yl) benzamide (4g)*

 White solid. m.p. 198-199 ℃; ^1^H NMR (400 MHz, DMSO-*d*6) δ: 8.83-8.84 (m, 1H, ArH), 8.53-8.54 (m, 1H, ArH), 8.05-8.08 (m, 2H, ArH), 8.00-8.03 (m, 1H, ArH), 7.58-7.60 (m, 1H, ArH), 7.45-7.53 (m, 3H, ArH), 3.73 (s, 2H, CH_2_), 2.49 (m, 8H, 4XNCH_2_), 2.25 (s, 3H, CH_3_); ^13^C NMR (100 MHz, DMSO-*d*6) δ: 172.6, 165.8, 157.9, 149.6, 149.1, 143.5, 136.3, 133.20, 132.5, 131.1, 129.2, 128.7, 124.1, 54.0, 53.6, 53.1, 51.3; HRMS calcd. for C_21_H_24_N_5_OS [M^+^+1] 394.1696, found 394.1699. 

*N-(5-((dimethylamino)methyl)-4-(pyridin-3-yl) thiazol-2-yl) isonicotinamid (4h)*

 white solid. m. p.201-202 ℃; ^1^H NMR (400 MHz, DMSO-*d*6) δ: 8.84-8.85 (m, 1H, ArH), 8.76-8.77 (m, 2H, ArH), 8.53-8.55 (m, 1H, ArH), 8.01-8.04 (m, 1H, ArH), 7.95-7.96 (m, 2H, ArH), 7.49-7.50 (m, 1H, ArH), 3.65 (s, 2H, ArCH_2_), 2.19 (s, 6H, CH_3_); ^13^C NMR (100 MHz, DMSO) δ: 164.4, 157.4, 150.9, 149.1, 143.1, 139.7, 136.2, 131.0, 128.6, 124.0, 122.3, 55.3, 45.5; HRMS calcd. for C_17_H_18_N_5_OS [M^+^+1] 340.1154, found 340.1158.

*N-(5-(morpholinomethyl)-4-(pyridin-3-yl) thiazol-2-yl) isonicotinamide (4i)*

Yellow solid. m. p.185-187 ℃; ^1^H NMR (400 MHz, DMSO-*d*6) δ: 8.86-8.87 (m, 1H, ArH), 8.79-8.80 (m, 2H, ArH), 8.65-8.66 (m, 1H, ArH), 8.09-8.12(m, 1H, ArH), 7.96-7.98 (m, 2H, ArH), 7.56-7.59 (m, 1H, ArH), 4.34 (s, 2H, ArCH_2_), 3.65-3.67 (m, 4H, 2XOCH_2_), 2.93-2.94 (m, 4H, 2XOCH_2_); ^13^C NMR (100 MHz, DMSO-*d*6) δ: 190.0, 164.8, 158.9, 150.9, 149.1, 148.9, 139.5, 137.8, 130.6, 127.2, 124.6, 122.3, 64.6, 51.8, 26.8; HRMS calcd. forC_20_H_19_N_5_O_2_S [M^+^+1] 382.4540, found 382.4544.

*N-(5-((dimethylamino)methyl)-4-(pyridin-3-yl) thiazol-2-yl)-2,3-dihydrobenzo[b] [1,4] dioxine-2-carboxamide (4j)*

 white solid. m. p.192-196 ℃; ^1^H NMR (400 MHz, DMSO-*d*6) δ: 8.80-8.81 (m, 1H, ArH), 8.52-8.53 (m, 1H, ArH), 7.97-8.00 (m, 1H, ArH), 7.43-7.47 (m, 1H, ArH), 6.96-6.99 (m, 1H, ArH), 6.81-6.87 (m, 3H, ArH), 5.11-5.13 (m, 1H, XOCH), 4.40-4.41 (m, 2H, XOCH_2_), 3.62 (s, 2H, ArCH_2_), 2.16 (s, 6H, CH_3_); ^13^C NMR (100 MHz, DMSO-*d*6) δ: 166.6, 156.4, 149.5, 149.0, 143.3, 143.0, 136.1, 130.9, 124.0, 122.3, 121.9, 117.7, 117.5, 72.4, 65.0, 55.4, 45.4; HRMS calcd. for C_20_H_21_N_4_O_3_S[M^+^+1] 397.4650, found 397.4652.

*N-(4-(pyridin-3-yl)-5-(pyrrolidin-1-ylmethyl) thiazol-2-yl)-2,3-dihydrobenzo[b] [1,4] dioxine-2-carboxamide (4k)*

 Yellow solid. m.p.181-182 ℃; ^1^H NMR (400 MHz, DMSO-*d*6) δ: 8.79-8.80 (m, 1H, ArH), 8.52-8.53 (m, 1H, ArH),7.96-7.99 (m, 1H, ArH), 7.44-7.47 (m, 1H, ArH), 6.95-6.97 (m, 1H, ArH), 6.80-6.88 (m, 3H, ArH), 5.16-5.17 (m,1H, XOCH), 4.36-4.45 (m, 2H, XOCH_2_), 3.69 (s, 2H, ArCH_2_), 3.50-3.52 (m, 4H, CH_2_), 2.36-2.38 (m, 4H, CH_2_); ^13^C NMR (100MHz, DMSO-*d*6) δ: 166.8, 149.5, 149.1, 143.4, 143.0, 136.1, 130.9, 127.5, 124.1, 122.3, 121.9, 117.6, 117.5, 72.3, 66.7, 55.4, 54.3, 53.6; HRMS calcd. forC_22_H_23_N_4_O_3_S[M^+^+1] 423.5030, found 423.5034.

*N-(5-(morpholinomethyl)-4-(pyridin-3-yl) thiazol-2-yl)-2,3-dihydrobenzo[b] [1,4] dioxine-2-carboxamide (4l)*

 Yellow solid. m. p. 177-179 ℃; ^1^H NMR (400 MHz, DMSO-*d*6) δ: 8.81-8.82 (m, 1H, ArH), 8.51-8.53 (m, 1H, ArH), 7.97-8.00 (m, 1H, ArH), 7.43-7.47 (m, 1H, ArH), 6.96-6.98 (m, 1H, ArH), 6.81-6.88 (m, 3H, ArH), 5.11-5.13 (m,1H, XOCH), 4.40-4.42 (m, 2H, XOCH_2_), 3.83 (s, 2H, ArCH_2_), 2.46-2.48 (m, 4H, CH_2_), 1.66-1.67 (m, 4H, CH_2_); ^13^C NMR (100MHz, DMSO-*d*6) δ:166.9, 156.3, 149.5, 149.0, 143.4, 143.0, 136.0, 131.0, 124.0, 122.3, 121.9, 117.7, 117.5, 72.3, 65.0, 55.4, 54.1, 23.7; HRMS calcd. forC_22_H_23_N_4_O_4_S[M^+^+1] 439.1362, found 439.1366.

*4-Methyl-N-(5-(morpholinomethyl)-4-(pyridin-4-yl) thiazol-2-yl) benzamide (4m)*

 White solid. m.p. 148-149℃; ^1^H NMR (600 MHz, DMSO-*d*6) δ: 12.67 (br s, 1H, NH), 8.65-8.67 (m, 2H, ArH), 8.01-8.03 (m, 2H, ArH), 7.68-7.69 (m, 2H, ArH), 7.34-7.37 (m, 2H, ArH), 3.81 (s, 2H, ArCH_2_), 3.59-3.61 (m, 4H, 2XOCH_2_), 2.49-2.50 (m, 4H, 2XNCH_2_), 2.33 (s, 3H, CH_3_); ^13^C NMR (125 MHz, DMSO-*d*6) δ: 165.6, 157.9, 150.4, 143.6, 143.5, 142.3, 129.7, 129.6, 129.1, 128.8, 123.3, 66.8, 54.4, 53.7, 21.6; HRMS calcd. for C_ 21_H_23_N_4_O_2_S [M^+^+1] 395.1536, found 395.1533.

*4-Methyl-N-(5-((4-methylpiperazin-1-yl) methyl)-4-(pyridin-4-yl) thiazol-2-yl) benzamide (4n)*

 White solid. m. p. 148-150 ℃; ^1^H NMR (400 MHz, DMSO-*d*6) δ: 12.62 (br s, 1H, NH), 8.65-8.66 (m, 2H, ArH), 8.01-8.03 (m, 2H, ArH), 7.67-7.69 (m, 2H, ArH), 7.34-7.36 (m, 2H, ArH), 3.80 (s, 2H, ArCH_2_), 2.33-2.39 (m, 8H, 4XNCH_2_), 2.23 (s, 3H, CH_3_); ^13^C NMR (100 MHz, DMSO-*d*6) δ: 165.6, 161.4, 157.8, 150.4, 143.4, 143.3, 142.4, 129.8, 129.7, 128.7, 123.3, 55.2, 54.1, 53.2, 46.1, 21.6; HRMS calcd. for C_22_H_26_N_5_OS [M^+^+1] 408.1853, found 408.1849.

*3,4-Dichloro-N-(5-((4-methylpiperazin-1-yl) methyl)-4-(pyridin-4-yl) thiazol-2-yl) benzamide (4o)*

Yellow solid. m.p. 201-203 ℃; ^1^H NMR (400 MHz, DMSO-*d*6) δ: 8.66-8.67 (m, 2H, ArH), 8.38-8.39 (m, 1H, ArH), 8.06-8.07 (m, 1H, ArH), 7.84-7.85 (m,1H, ArH), 7.67-7.68 (m, 2H, ArH), 3.83 (s, 2H, ArCH_2_), 2.49 (s, 8H, 2XNCH_2_), 2.27 (s, 3H, CH_3_); ^13^C NMR (100 MHz, DMSO-*d*6) δ: 163.8, 157.5, 150.5, 147.2, 142.2, 136.0, 132.9, 132.5, 132.1, 131.5, 130.7, 129.0, 123.3, 66.8, 60.3, 54.4, 53.7; HRMS calcd. for C_21_H_22_Cl_2_N_5_OS [M^+^+1] 462.0917, found 462.0911.

*3,4-Dichloro-N-(5-(morpholinomethyl)-4-(pyridin-4-yl) thiazol-2-yl) benzamide (4p)*

White solid. m.p.163-164 ℃; ^1^H NMR (400 MHz, DMSO-*d*6) δ: 8.67-8.68 (m, 2H, ArH), 8.38-8.39 (m, 1H, ArH), 8.06-8.07 (m, 1H, ArH), 7.84-7.85 (m,1H, ArH), 7.67-7.68 (m, 2H, ArH), 3.83 (s, 2H, ArCH_2_), 3.60 (s, 4H, 2XOCH_2_), 2.49 (s, 4H, 2XNCH_2_); ^13^C NMR (100 MHz, DMSO-*d*6) δ: 163.7, 157.6, 150.4, 143.4, 142.2, 136.0, 133.0, 132.1, 131.5, 130.7, 130.3, 129.0, 123.3, 54.8, 53.9, 52.5; HRMS calcd. for C_20_H_19_Cl_2_N_4_O_2_S [M^+^+1] 449.0600, found 449.0597.

*N-(5-((4-Methylpiperazin-1-yl) methyl)-4- (pyridin-4-yl) thiazol-2-yl) isonicotinamide (4q)*

White solid. m.p. 203-205 ℃; ^1^H NMR (400 MHz, DMSO-*d*6) δ: 12.93 (br s, 1H, NH), 8.76-8.77 (m, 2H, ArH), 8.61-8.62 (m, 2H, ArH), 7.94-7.96 (m, 2H, ArH), 7.62-7.63 (m, 2H, ArH), 3.72 (s, 2H, ArCH_2_), 2.39 (m, 4H, CH_2_), 1.46-1.47 (m, 4H, CH_2_), 1.33-1.34 (m, 2H, CH_2_); ^13^C NMR (100 MHz, DMSO-*d*6) δ: 164.4, 157.3, 150.9, 150.4, 143.1, 142.2, 139.6, 131.3, 123.3, 122.3, 54.9, 54.6, 26.1, 24.3; HRMS calcd. for C_20_H_22_N_6_OS [M^+^+1] 380.1467, found 380.1465.

*N-(4-(Pyridin-4-yl)-5-(pyrrolidin-1-ylmethyl) thiazol-2-yl) isonicotinamide (4r)*

White solid; m.p. 192-195℃; ^1^H NMR (400 MHz, DMSO-*d*6) δ: 8.76-8.77 (m, 2H, ArH), 8.61-8.63 (m, 2H, ArH), 7.94-7.96 (m, 2H, ArH), 7.64-7.65 (m,1H, ArH), 3.90 (s, 2H, ArCH_2_), 2.53 (s, 4H, CH_2_), 1.69 (s, 2H, CH_2_); ^13^C NMR (100 MHz, DMSO-*d*6) δ: 164.5, 160.8, 151.0, 150.9, 150.5, 143.3, 142.2, 139.7, 123.2, 122.3, 54.2, 51.9, 23.8; HRMS calcd. for C_19_H_20_N_5_OS [M^+^+1] 366.1383, found 366.1391.

*N-(5-(morpholinomethyl)-4-(pyridin-4-yl) thiazol-2-yl) isonicotinamide (4s)*

White solid. m.p.175-178℃; ^1^H NMR (400 MHz, DMSO-*d*6) δ: 8.77-8.78(m, 2H, ArH), 8.62-8.64 (m, 2H, ArH), 7.95-7.96 (m, 2H, ArH), 7.64-7.65 (m, 2H, ArH),3.80 (s, 2H, ArCH_2_), 3.55-3.58 (m, 4H, 2XOCH_2_), 2.45-2.46 (m, 4H, 2XOCH_2_); ^13^C NMR (100MHz, DMSO-d6) δ: 164.50, 157.38, 150.99, 150.98, 150.46, 142.14, 139.59, 129.91, 123.31, 122.30, 66.76, 54.45, 53.75; HRMS calcd. forC_19_H_20_N_5_O_2_S[M^+^+1] 382.1259, found 382.1263.

*N-(5-(Morpholinomethyl)-4-(pyridine-4-yl) thiazol-2-yl) nicotinamide (4t)*

White solid. m.p.178-180 ℃; ^1^H NMR (400 MHz, DMSO-*d*6) δ: 9.17-9.18 (m, 1H, ArH), 8.74-8.75 (m, 1H, ArH), 8.62-8.63 (m, 2H, ArH), 7.37-7.39 (m,1H, ArH), 7.55-7.56 (m, 2H, ArH), 7.52-7.53 (m, 1H, ArH), 3.78 (s, 2H, ArCH_2_), 3.56 (s, 4H, 2XOCH_2_), 2.44 (s, 4H, 2XNCH_2_); ^13^C NMR (100 MHz, DMSO-*d*6) δ: 164.5, 157.5, 153.5, 150.4, 149.7, 143.5, 142.2, 136.4, 129.6, 128.4, 124.1, 123.3, 66.7, 54.4, 53.7; HRMS calcd. for C_19_H_20_N_5_O_2_S [M^+^+1] 382.1332, found 382.1329.

*N-(5-(Piperidin-1-ylmethyl)-4-(pyridin-4-yl) thiazol-2-yl) nicotinamide (4u)*

White solid. m.p.169-171 ℃. ^1^H NMR (400 MHz, DMSO-*d*6) δ: 9.18-9.20 (m, 1H, ArH), 8.75-8.77 (m, 1H, ArH), 8.62-8.63 (m, 2H, ArH), 7.38-7.40 (m, 1H, ArH), 7.65-7.66 (m, 2H, ArH), 7.54-7.56 (m, 1H, ArH), 3.76 (s, 2H, ArCH_2_), 2.42 (s, 4H, 2XNCH_2_), 1.48-1.50 (m, 4H, 2CH_2_), 1.33-1.34 (m, 2H, CH_2_); ^13^C NMR (100MHz, DMSO-*d*6) δ: 164.5, 153.5, 150.4 (2C), 149.7(2C), 142.5, 136.4 (2C), 128.5, 124.1, 123.4, 54.9, 54.6, 26.0, 24.3; HRMS calcd. for C_20_H_22_N_5_OS [M^+^+1] 380.1540, found 380.1538.

*N-(5-(morpholinomethyl)-4-(pyridin-4-yl) thiazol-2-yl)-2,3-dihydrobenzo[b] [1,4] dioxine-2-carboxamide (4v)*

yellow solid; m. p.178-180℃; ^1^H NMR (400 MHz, CD_3_OD) δ: 8.55-8.57 (m, 2H, ArH), 7.74-7.76 (m, 2H, ArH), 7.05-7.07 (m, 1H, ArH), 6.85-6.89 (m, 3H, ArH), 5.01-5.04 (m,1H, XOCH), 4.40-4.43 (m, 2H, XOCH_2_), 3.78 (s, 2H, ArCH_2_), 3.66-3.68 (m, 4H, CH_2_), 2.49-2.50 (m, 4H, CH_2_); ^13^C NMR (100MHz, CD_3_OD) δ: 166.8, 156.3, 148.8, 143.3, 141.9, 130.0, 123.4, 121.9, 121.7, 117.3, 117.0, 72.8, 66.6, 64.4, 54.0, 53.3; HRMS calcd. For C_22_H_23_N_4_O_4_S [M^+^+1] 439.1362, found 439.1364.

*Enzyme-based ROCK II inhibitory activity studies*

The enzyme-based ROCK II inhibitory activity assay was performed using the Rho kinase assay kit (CY-1160, Cycle, Nagoya, Japan). All operation was guided by the manufacturer’s instructions. Briefly, the compounds at 10 µM were pre-incubated in a system wherein ROCK II (0.02 ng/µL) phosphorylates the myosin-binding subunit (MBS) of kinase substrate pre-absorbed onto the microplate in the presence of Mg^2+ ^and ATP. Then, the system was washed after incubation at 30 ^◦^C for 30 min. 100 µL of antibody was added to the wells and then incubated for 30 min at room temperature. 100 µL of substrate reagent and 100 µL stop solution were successively added to each well. The plates were read on the Viewlux in HTRF mode within 30 min.

*Molecular docking*

Molecular docking was performed using the Surflex-Dock module in Sybyl 2.0 software package. The crystal structure of human ROCK II (PDB code: 4L6Q) was download from RCSB Protein Data Bank. Before the docking process, the natural co-crystallized ligand was extracted, and water molecules were removed from the crystal structure, H atoms were added, and side chains were fixed during protein preparation. Subsequently, the protein was prepared using the Biopolymer module implemented in Sybyl. Protein structure minimization was performed by applying the Tripos force field, and partial atomic charges were calculated by the Gasteiger-Huckel method. All parameters were set to default values.

**Scheme 1:**
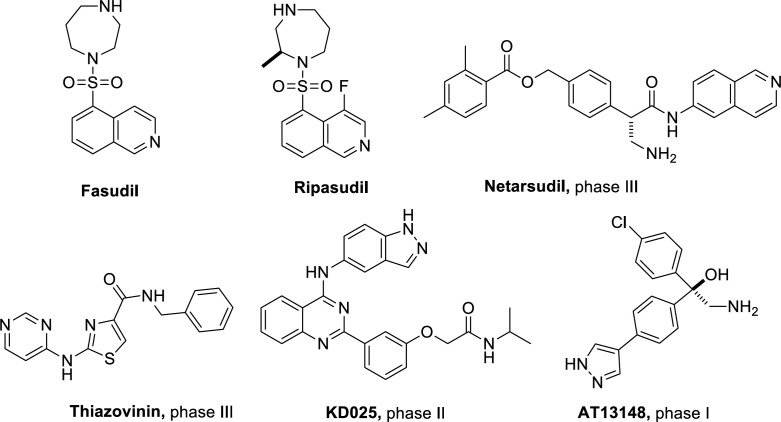
Synthesis of 4-aryl-5-aminomethyl-thiazole-2-amines. Reagents and conditions: (a) 1) Br_2_, AcOH, 48% HBr, 2) thiourea, EtOH, reflux; (b) EDCI, HOBt, R_1_C_6_H_5_COOH, DIPEA, DMF, r.t.; (c) HNR_2_R_3_, (CH_2_O) n, AcOH, 70 ºC

**Figure 3:**
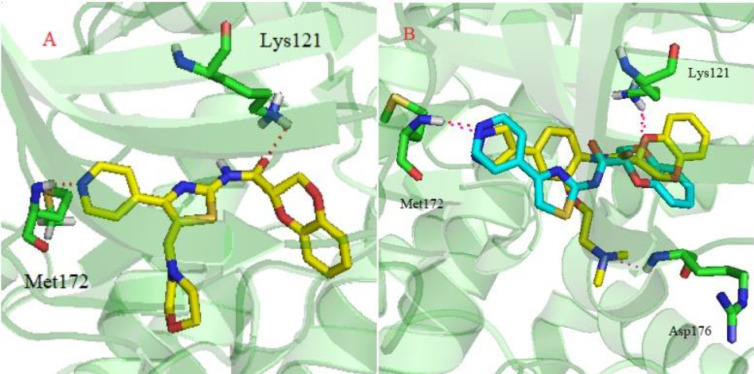
(A) Predicted binding mode of compound** 4v** (yellow) with ROCK II (PDB ID:4L6Q); (B) Predicted binding mode of compound** I** (yellow) and **II** (blue) with ROCK II (PDB ID:4L6Q). The residues in ROCK II are shown in green sticks. Hydrogen bonds are shown as red dashed lines

**Table 1:** ROCK II inhibition of 4-aryl**-**5-aminomethyl-thiazol-2-amines

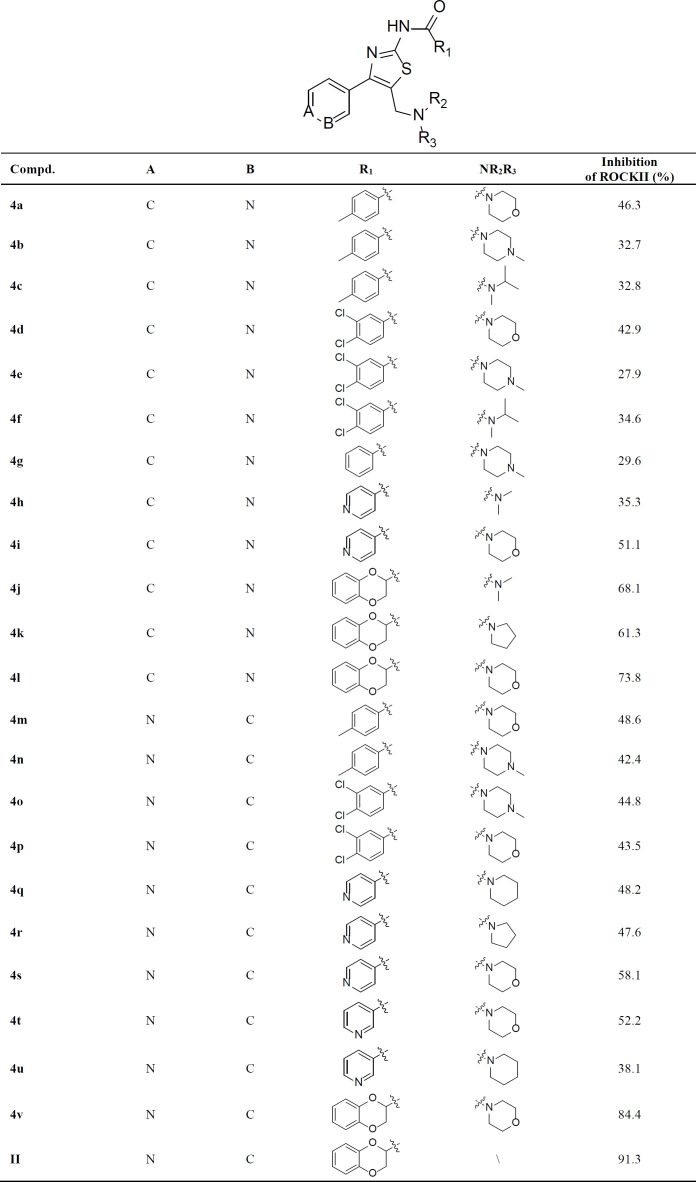

Percentage inhibition of **4a-4v** against ROCK II under the concentration of 10 µM.

**Table 2:** The IC_50_ values of compounds against ROCK II

**Compd.**	**ROCK II (µM)**	**Compd.**	**ROCK II (µM)**
**4k**	1.56 ± 0.23	**4t**	9.12 ± 2.46
**4i**	9.83 ± 1.45	**4l**	1.02 ± 0.07
**4v**	0.02 ± 0.01	**4j**	0.96 ± 0.12
**4s**	8.56 ± 067	**II**	0.01 ± 0.003

## Results and Discussion

*Chemistry*

The synthesis of 4-aryl-5-aminoalkyl-thiazole-2-amine **4 **was achieved in three steps, as shown in Scheme 1. Firstly, 4-aryl-thiazol-2-amine **2** was prepared by a modified procedure reported by Green and co-workers (27). This synthesis involved α-bromination of commercially available 3/4-acetylpyridine **1** with bromine and then cyclization reaction of bromoketone with thiourea. Secondly, amine **2** reacted with appropriate aromatic acids to afford the corresponding amide **3** in moderate yield. There were six (hetero)aromatic acids at this stage, including nicotinic acid, isonicotinic acid, and 1,4-benzodioxan-2-carboxylic acid, which were chosen in this step. Finally, the aminomethyl group was installed by treating compound **3** with paraformaldehyde and appropriate amines through a Mannich reaction. 

*Enzyme-based ROCK II inhibitory activity studies*

The inhibitory activities of compounds **4a**-**4v** on ROCK II were screened by enzyme-linked immunosorbent assay (ELISA), and compound **II **(Figure 1) was chosen as the control (28). All compounds were initially evaluated for their percentage inhibition against ROCK II with a ROCK II assay kit (CY-1160, Cycle, Japan). Most of the compounds showed a certain ROCK II inhibitory effect at the concentration of 10 µM, with the percentage inhibition range from 27% to 84% (Table 1). Then, compounds **4k**,** 4j**,** 4l**, **4s**, **4t**, **4v** were further evaluated in full concentration-response plots, and the IC_50_ values were shown in Table 2. Compared with the control compound **II**, these compounds were a little less potent, with the IC_50_ value range from 0.02 to 9.83 µM. The most potent compound **4v** exhibited ROCK II inhibition with the IC_50 _value of 20 nM. These preliminary biological assays partially justified our design, and further optimization will lead to more potent candidates. 

Based on the given data, the preliminary structure-activity relationship (SAR) could be summarized as follows. Firstly, the aromatic acids (R_1_ group), compounds with 1,4-benzodioxan-2-carboxylic acid, showed better activities than those with other acids. For example, compound **4v **(IC_50_ = 0.02 µM) was more potent than compound **4t** (IC_50_ = 9.12 µM) and **4p**. Compound **4l** (IC_50_ = 1.02 µM) was more potent than compound **4i** (IC_50_ = 9.83 µM), and **4a**. Secondly, due to the alkyl group on the aminomethyl side chain (R_2_, R_3 _group), compounds with the morpholine group generally were more potent than compounds with other groups (dimethylamine, isopropylamine, *N*-methyl piperazine, piperidine, pyrrolidine). For example, compound **4s **(IC_50_ = 8.56 µM) was more potent than compound **4q** and **4r**. Compound **4i** (IC_50_ = 9.83 µM) was more potent than compound **4h**. However, compounds **4j **(IC_50_ = 0.96 µM), **4k **(IC_50_ = 1.56 µM), and **4l **(IC_50_ = 1.02 µM) were exception, and they almost exhibited the same potency. It might suggest that 1,4-benzodioxan-2-carboxylic acid plays a critical role in maintaining the ROCK II inhibitory activity. Thirdly, for the position *N* in pyridine, compounds with 4-position substitution were generally more potent than compounds with 3-position substitution. For example, compound **4o **was more potent than compound **4e**, and compound **4s** was more potent than compound **4i**. In summary, the most potent compound **4v** showed inhibitory activity with an IC_50 _value of 20 nM.

*Molecular Docking Studies*

To further identify the possible binding modes of our synthesized ROCK II inhibitors, molecular docking was performed according to the general protocol. The crystal structure of ROCK II was taken from the RSCB Protein Data Bank (PDB code: 4L6Q) (29). Two lead compounds, **I** and **II,** and the most potent compound, **4v, **were chosen for this docking study using Sybyl-X2.0 software. As shown in Figure 3, compound **4v** displayed a similar bonding mode with compounds **I** and** II**. There was a key H-bonding interaction between Met172 and the position *N* in the pyridine or pyrazole ring. The amide groups were predicted to form another H-bonding interaction with Lys121. Both H-bonds play a pivotal role in stabilizing the orientation and conformation of ROCK inhibitors. The aminoethoxyl side chain of compound **I **was predicted to have an H-bonding interaction with Asp176. However, this H-bonding interaction was absent in compound **4v**. This might partially explain why compound **4v** was less potent than the lead compounds. Based on the above observations, further optimization would provide potent ROCK II inhibitor candidates.

## Conclusion

In a word, a series of 4-aryl-5-aminomethyl-thiazoleamines were designed and synthesized as a new class of ROCK II inhibitors. Then, in vitro ROCK II inhibitory activity assay showed that **4v** was the most potent compound, with the IC_50_ value of 20 nM. It might represent a promising lead compound for the further development of novel ROCK II inhibitors. Further optimizations of this scaffold, including the *in vivo* studies and biological evaluation in cell-based assays, will be reported in due course. 

## Supporting information

The data that supports the findings of this study are available in the supplementary material of this article.
