# Supplementary material for: The Design, Synthesis and Evaluation of Rho-kinase Inhibitory Activity of 4-aryl-thiazole-2-amines
Source: Iran J Pharm Res. 2021 Summer;20(3):121–31. doi: 10.22037/ijpr.2020.114468.14866 (PMC8653655; doi:10.22037/ijpr.2020.114468.14866)
Supplement: Supplementary file 1 [file ijpr-20-121-s001.pdf]

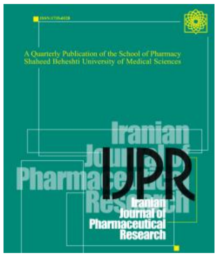

Supplementary Materials for

The Design, Synthesis and Evaluation of Rho-kinase Inhibitory Activity of 4-aryl-thiazole-2-amines

Linan Wang, Ben Ouyang, Meixia Fan, Junhui Qi and Lei Yao*

*To whom correspondence should be addressed. E-mail: yaoleiytu@163.com

Volume 20, Issue 3 (Summer 2021)

This PDF file includes:
Figure S1-S23

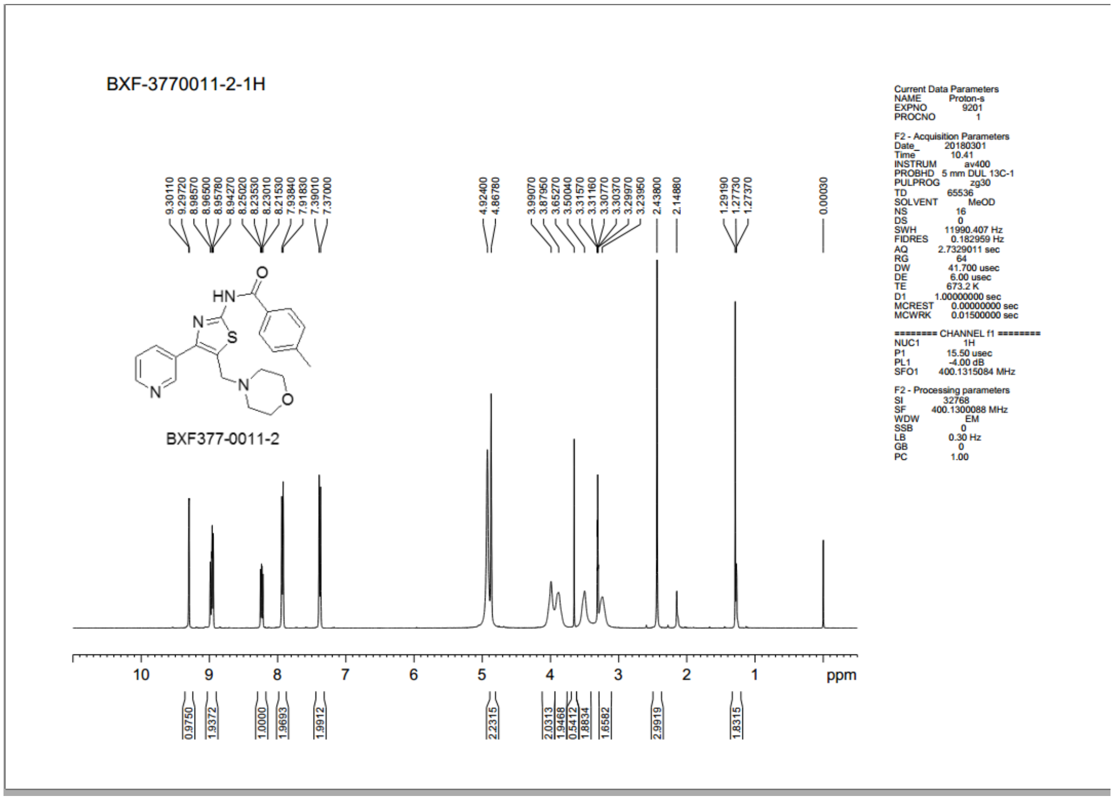

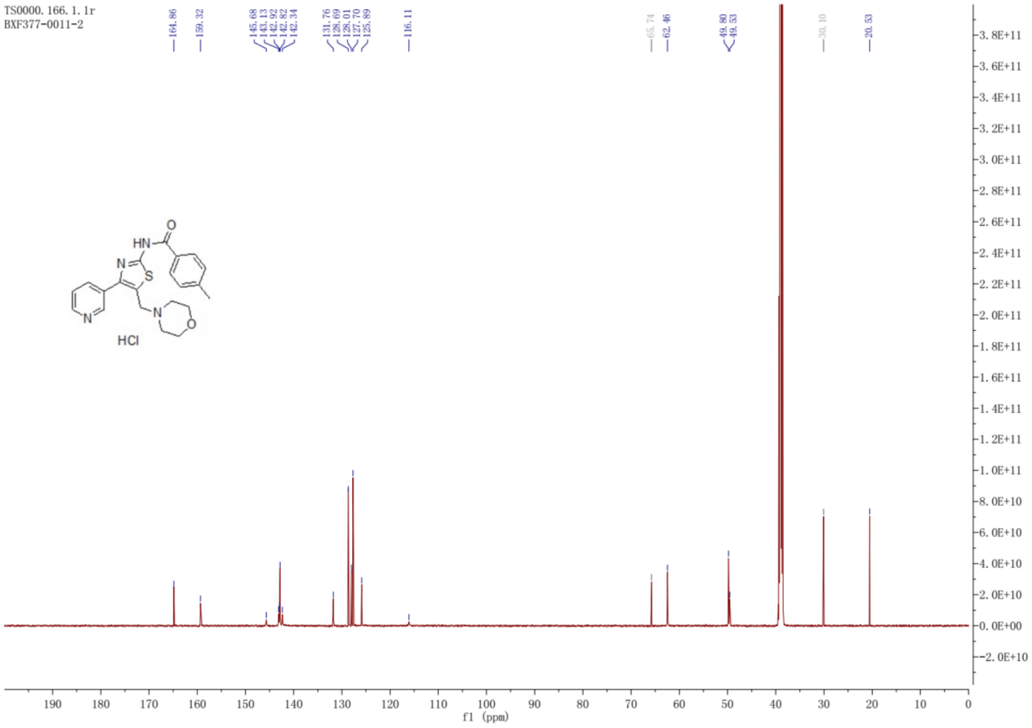

Figure S1. ^{1}H-NMR and ^{13}C-NMR spectrum of **4a**.

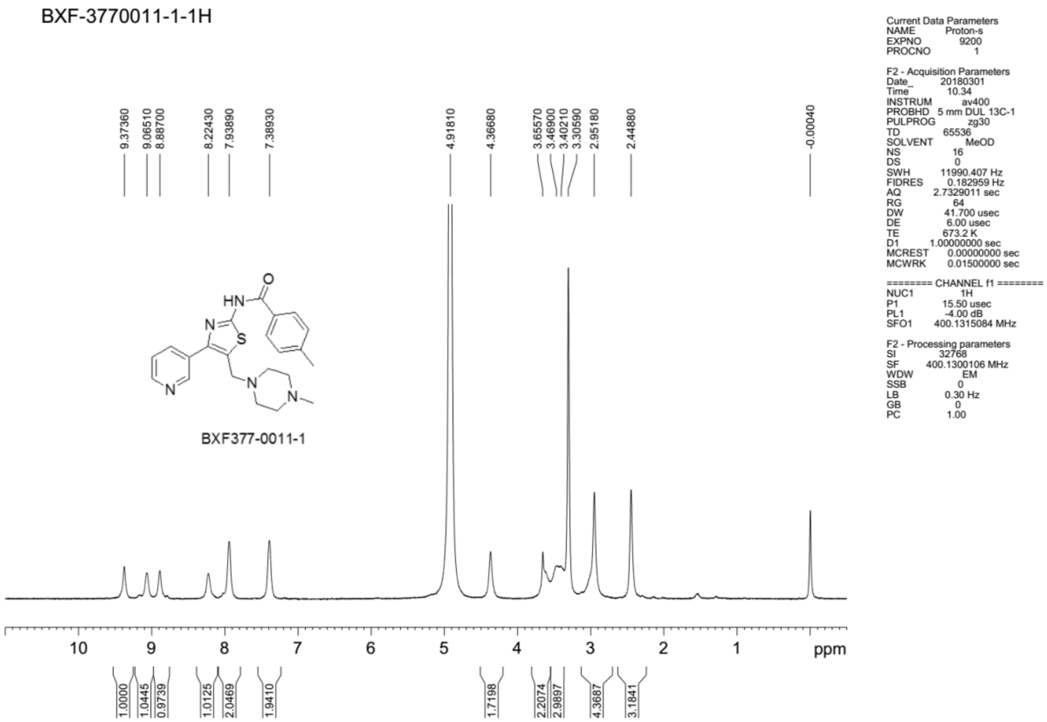

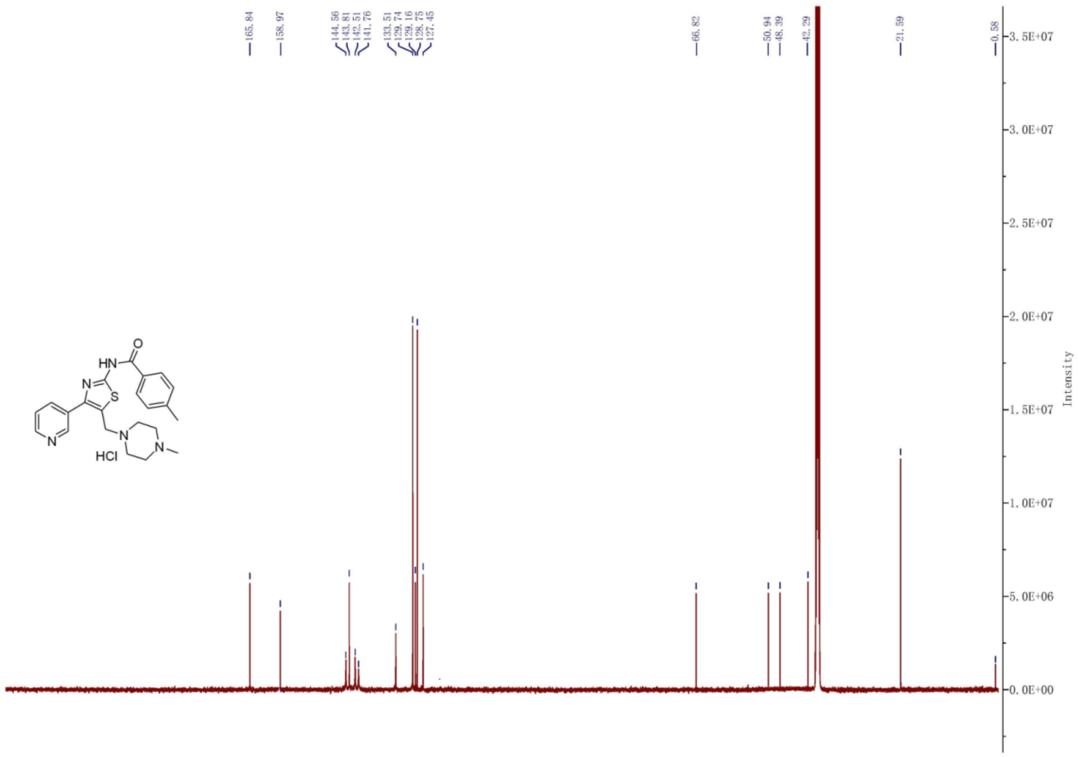

Figure S2. ^{1}H-NMR and ^{13}C-NMR spectrum of **4b**.

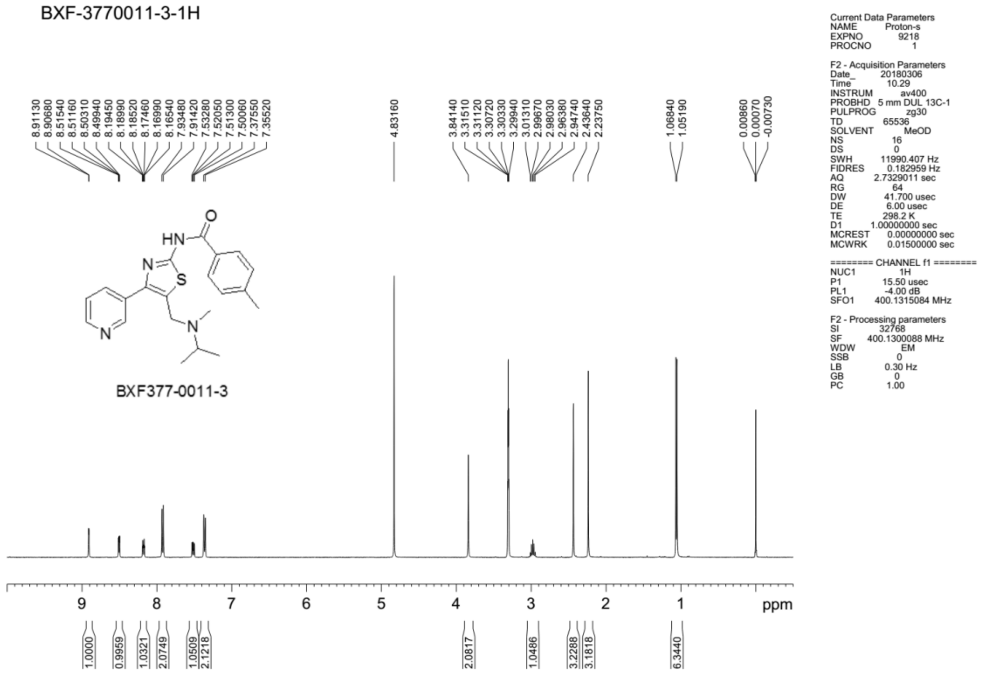

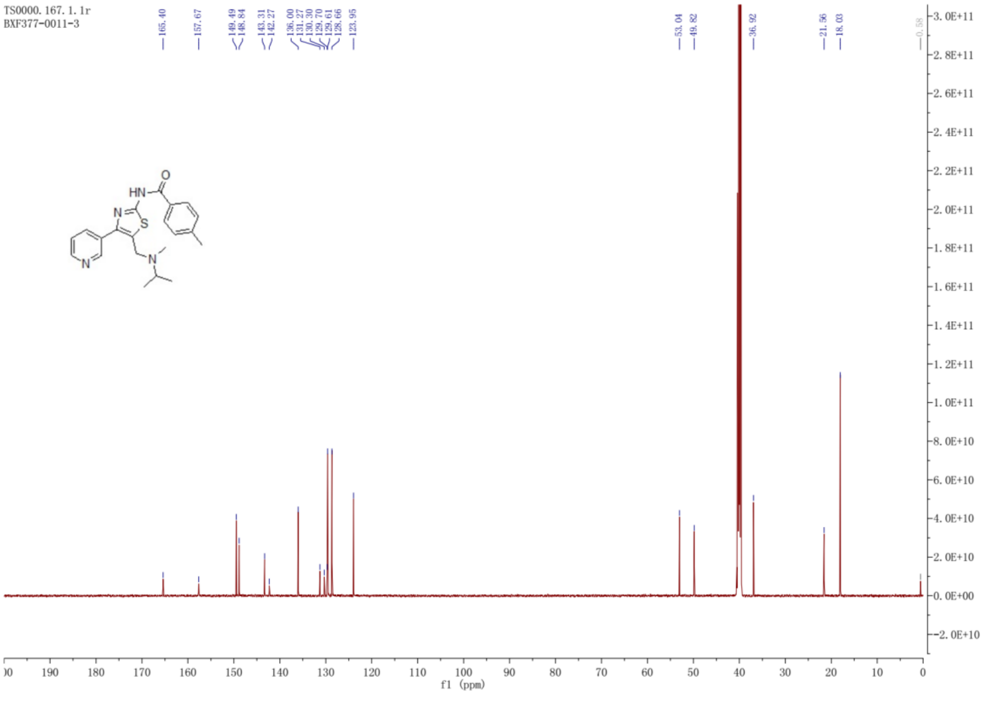

Figure S3. ^{1}H-NMR and ^{13}C-NMR spectrum of **4c**.

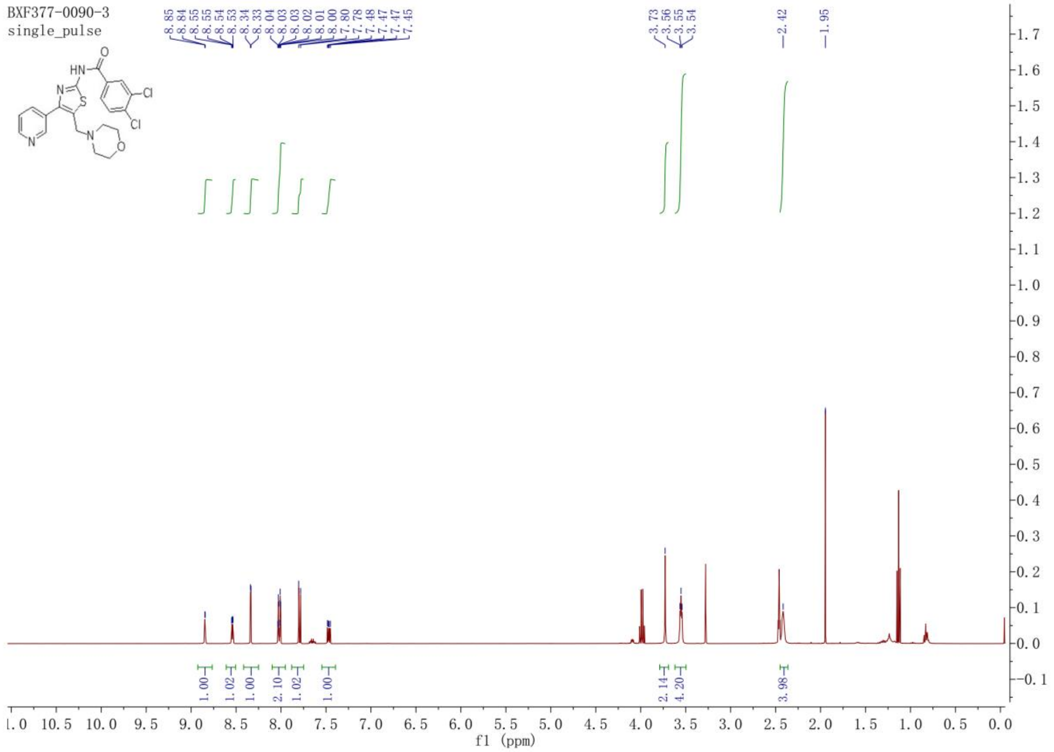

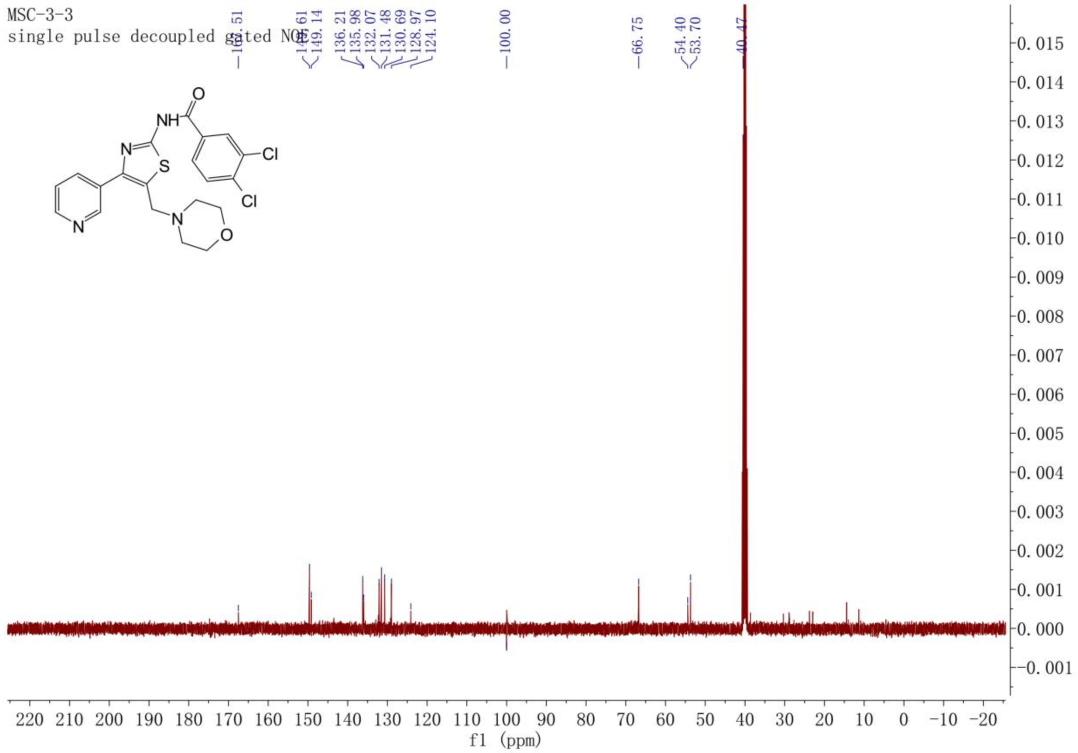

Figure S4. ^{1}H-NMR and ^{13}C-NMR spectrum of **4d**.

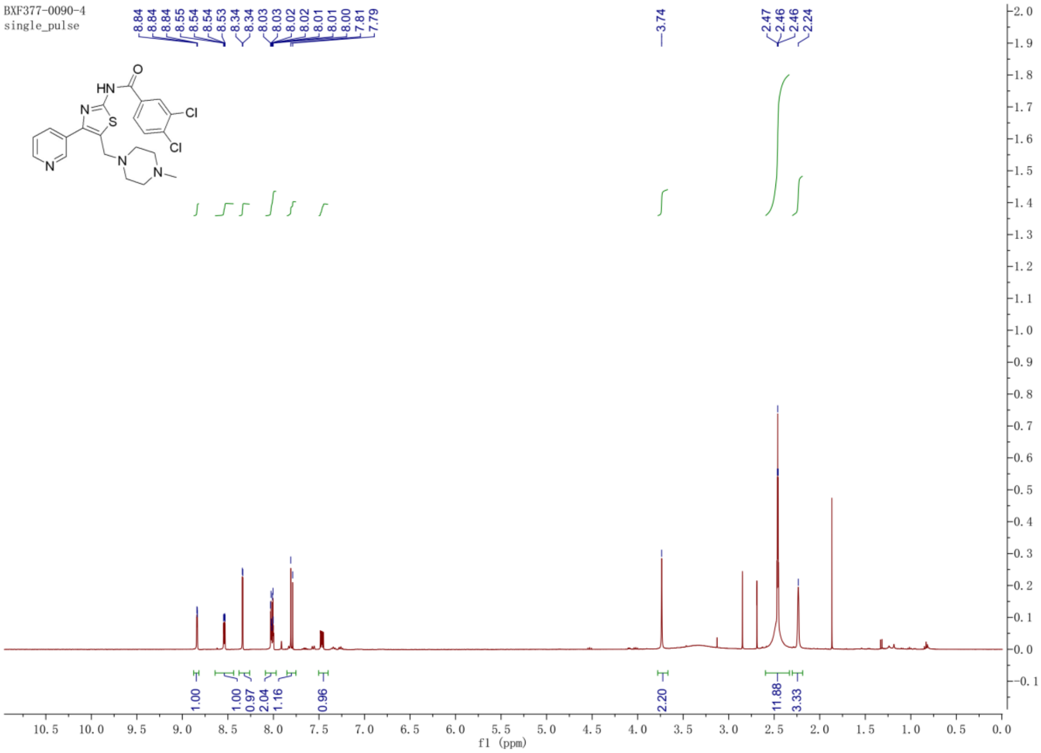

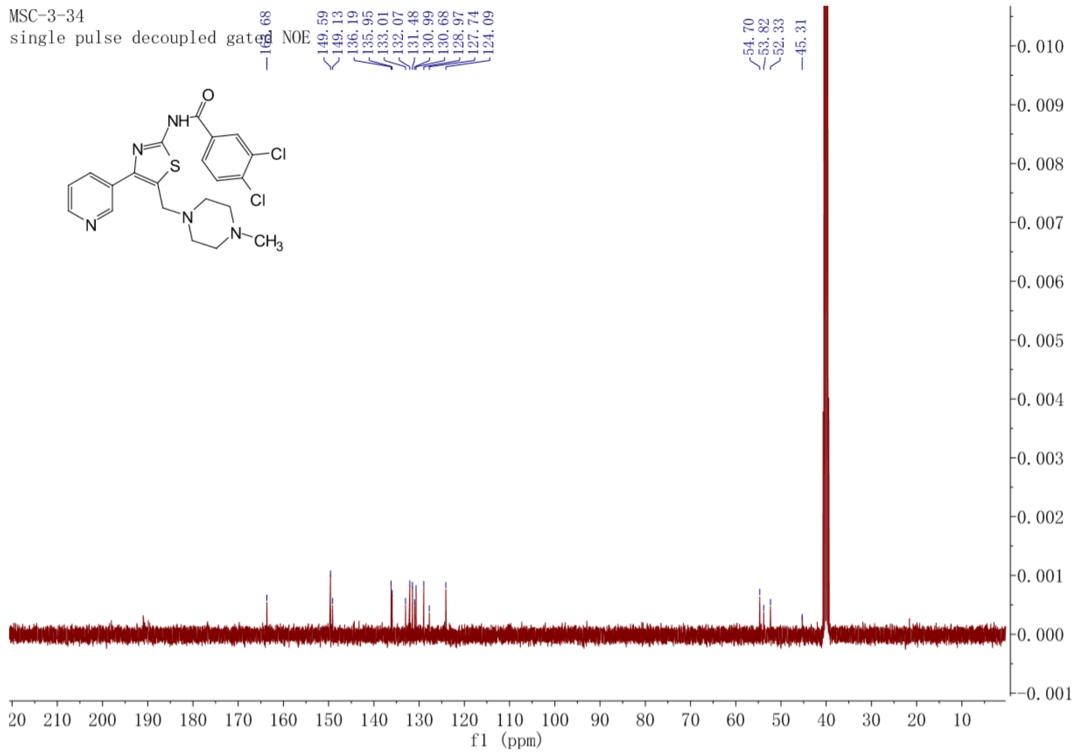

Figure S5. ^{1}H-NMR and ^{13}C-NMR spectrum of **4e**.

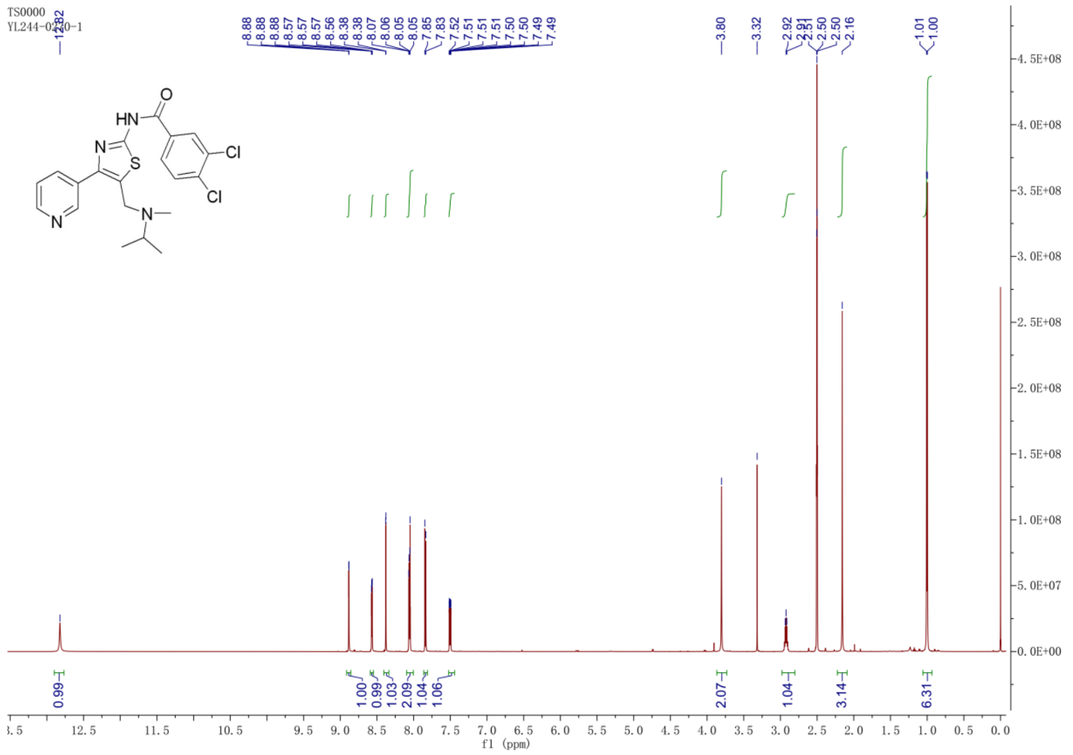

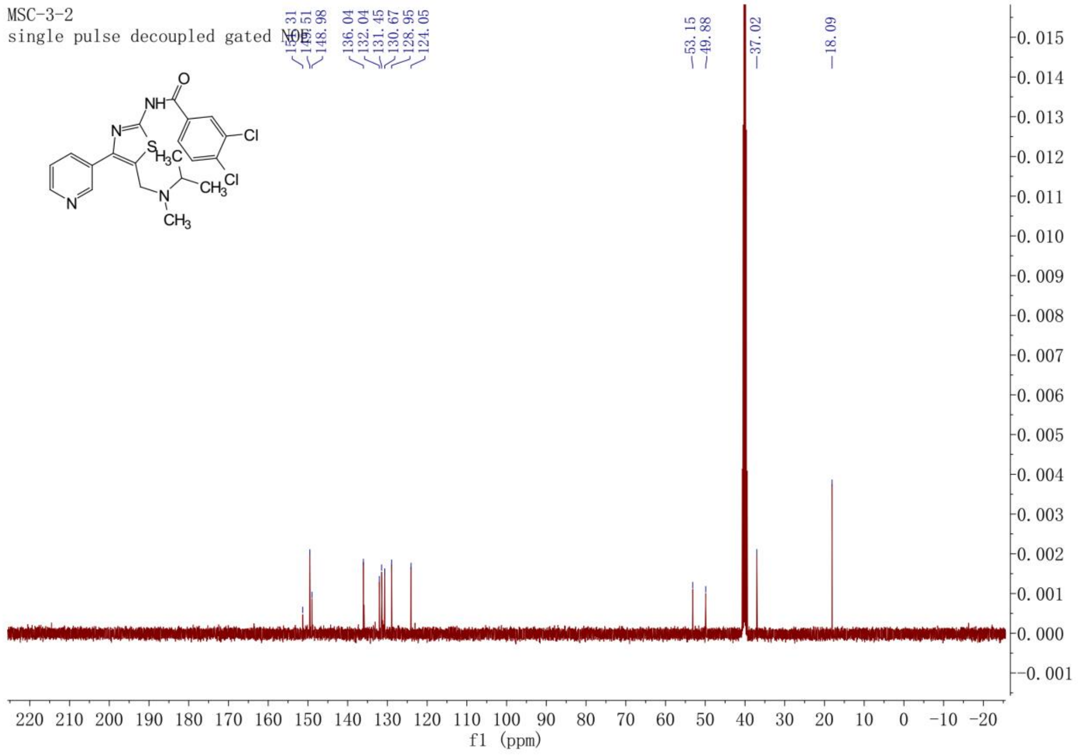

Figure S6. ^{1}H-NMR and ^{13}C-NMR spectrum of **4f**.

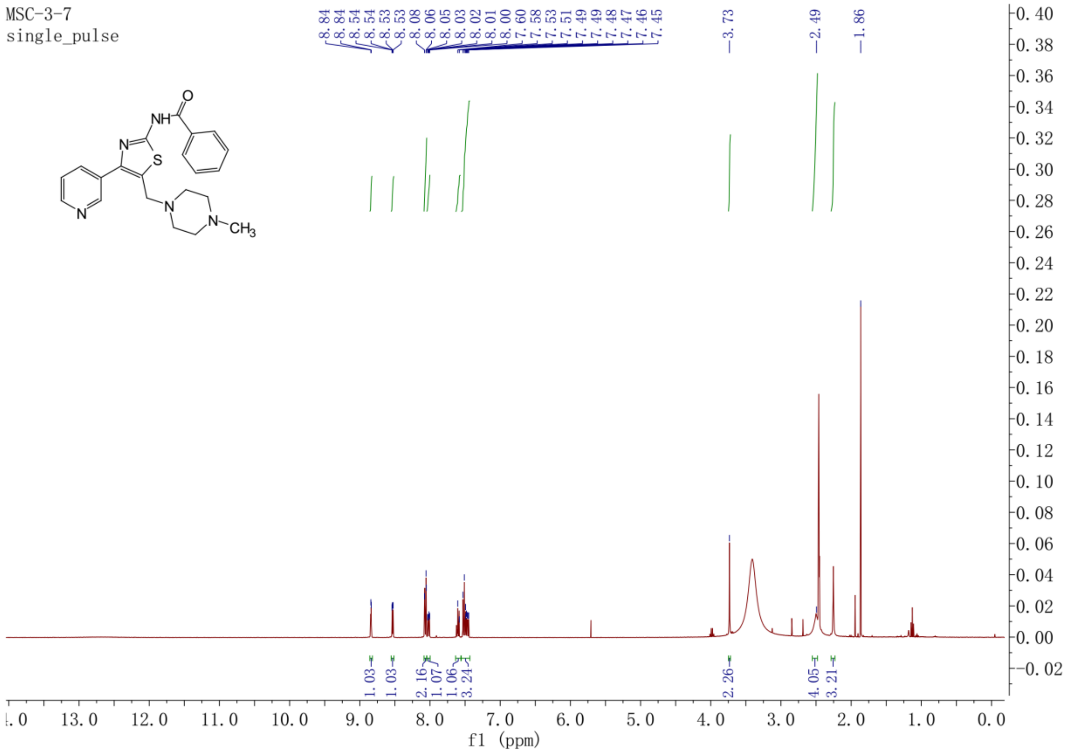

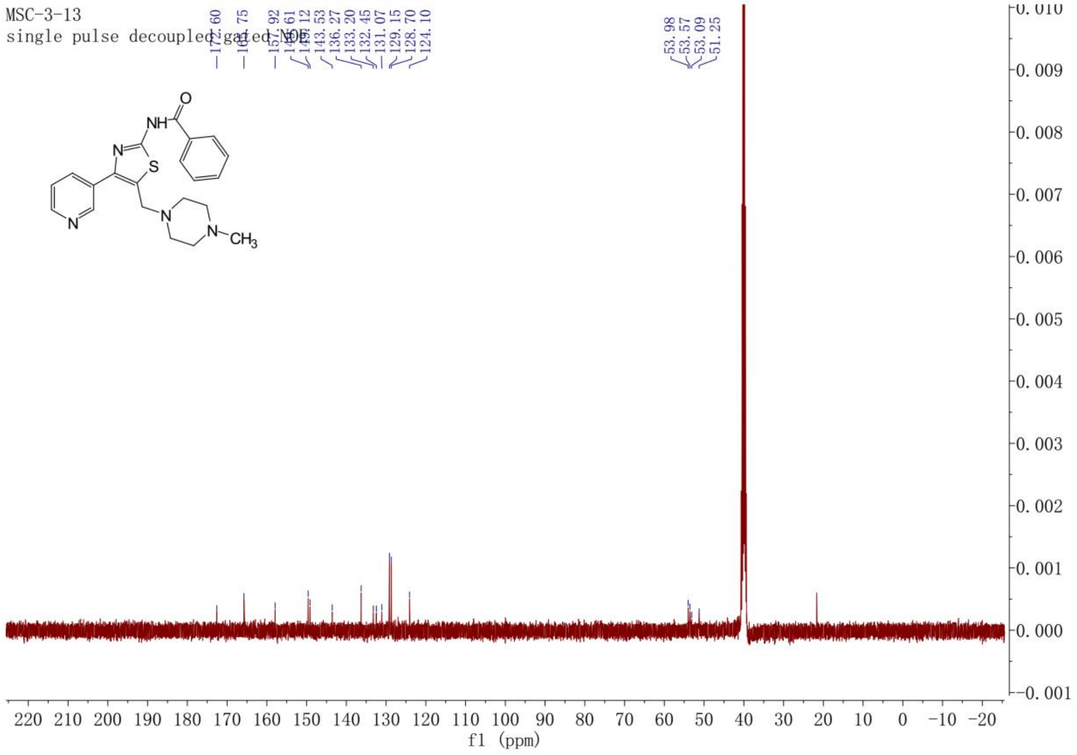

Figure S7. ^{1}H-NMR and ^{13}C-NMR spectrum of **4g**.

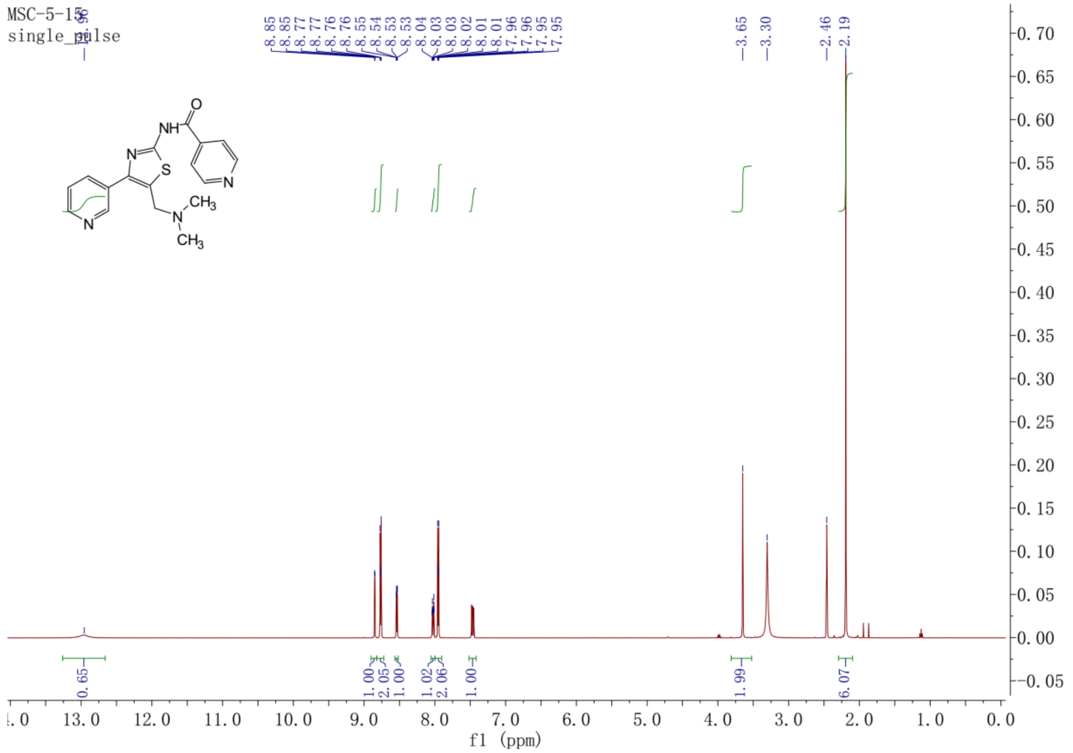

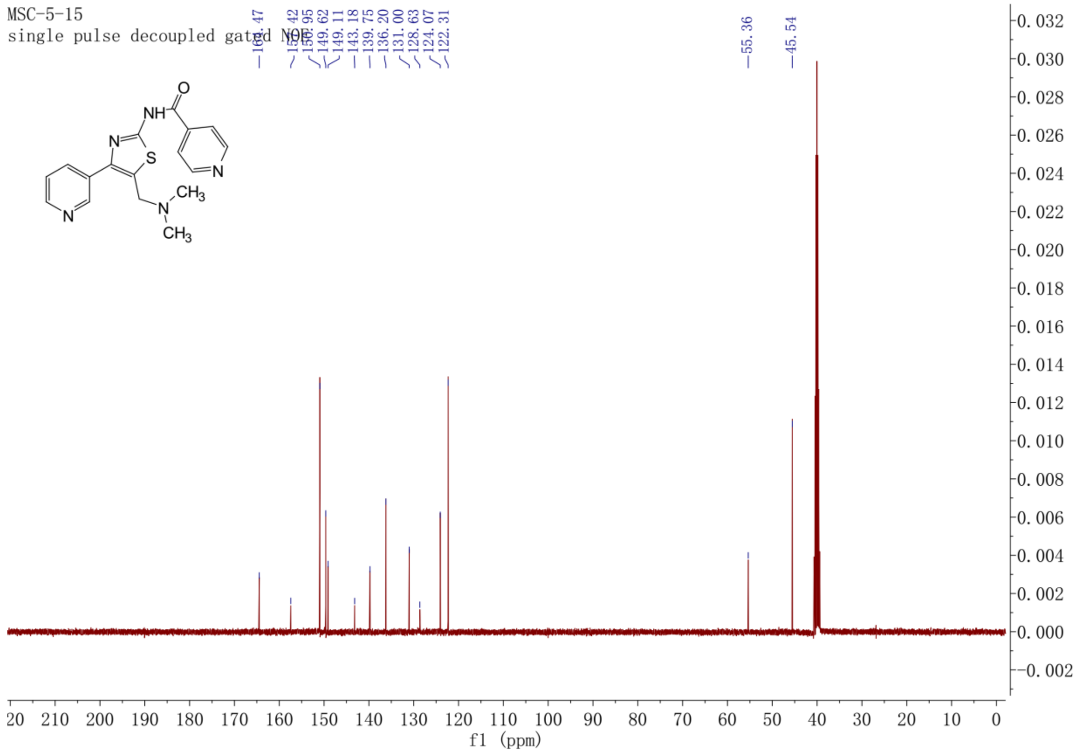

Figure S8. ^{1}H-NMR and ^{13}C-NMR spectrum of **4h**.

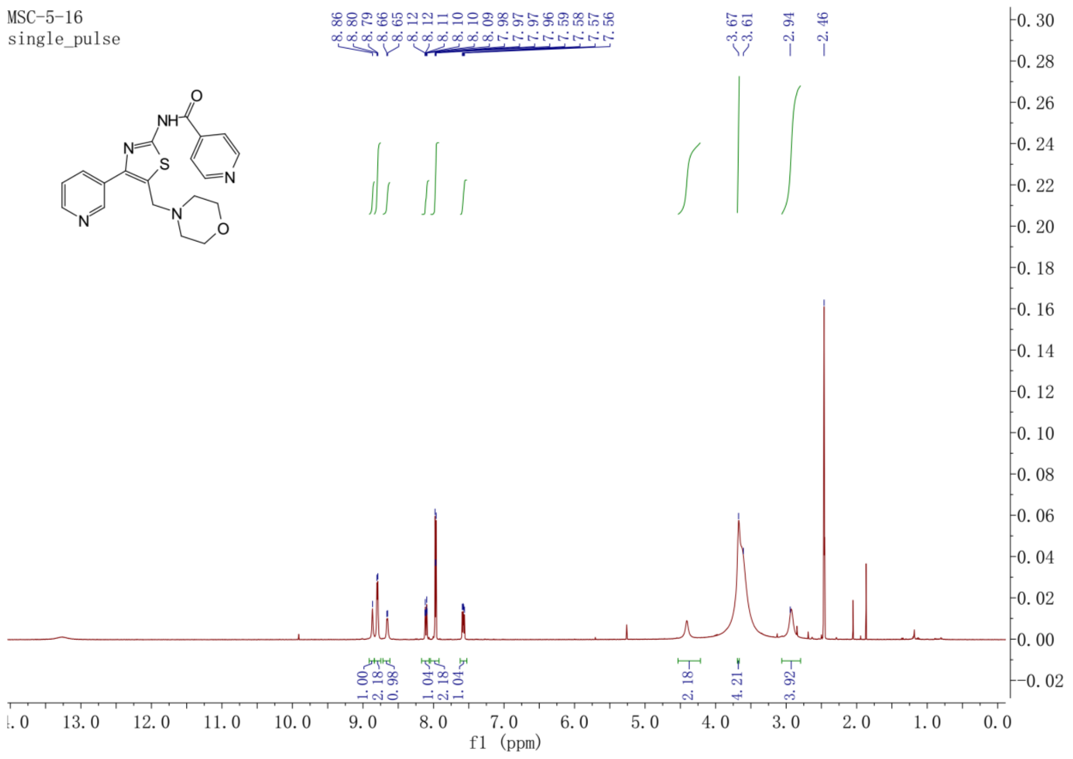

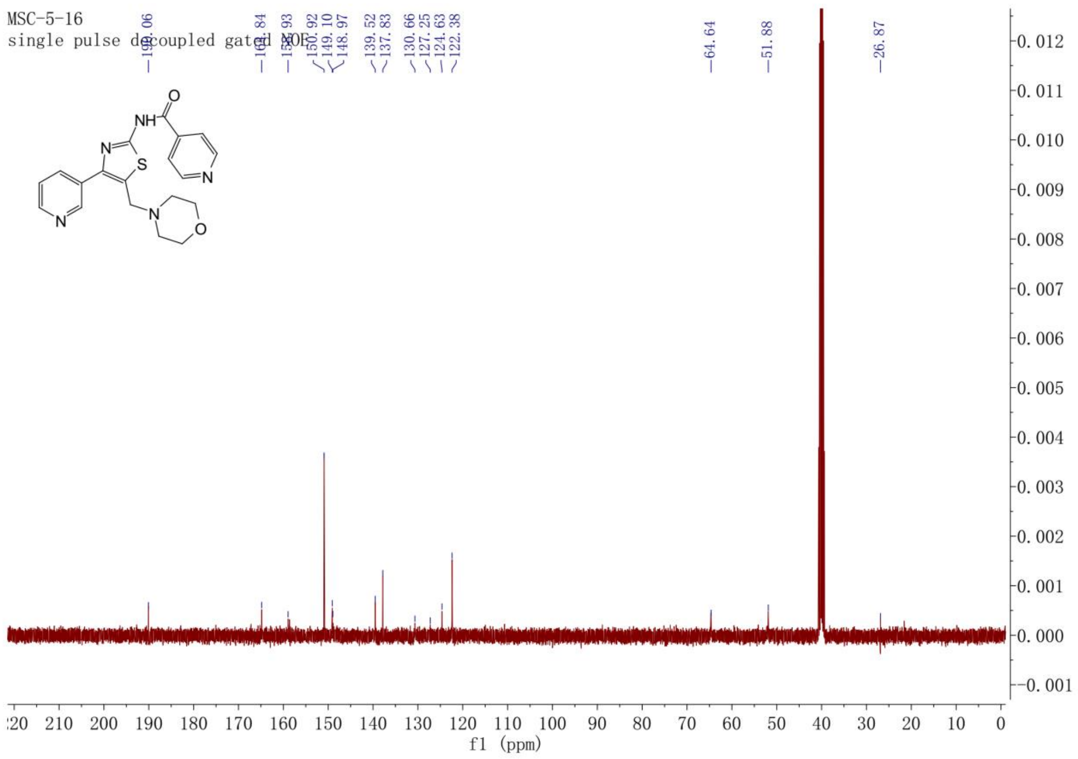

Figure S9. ^{1}H-NMR and ^{13}C-NMR spectrum of **4i**.

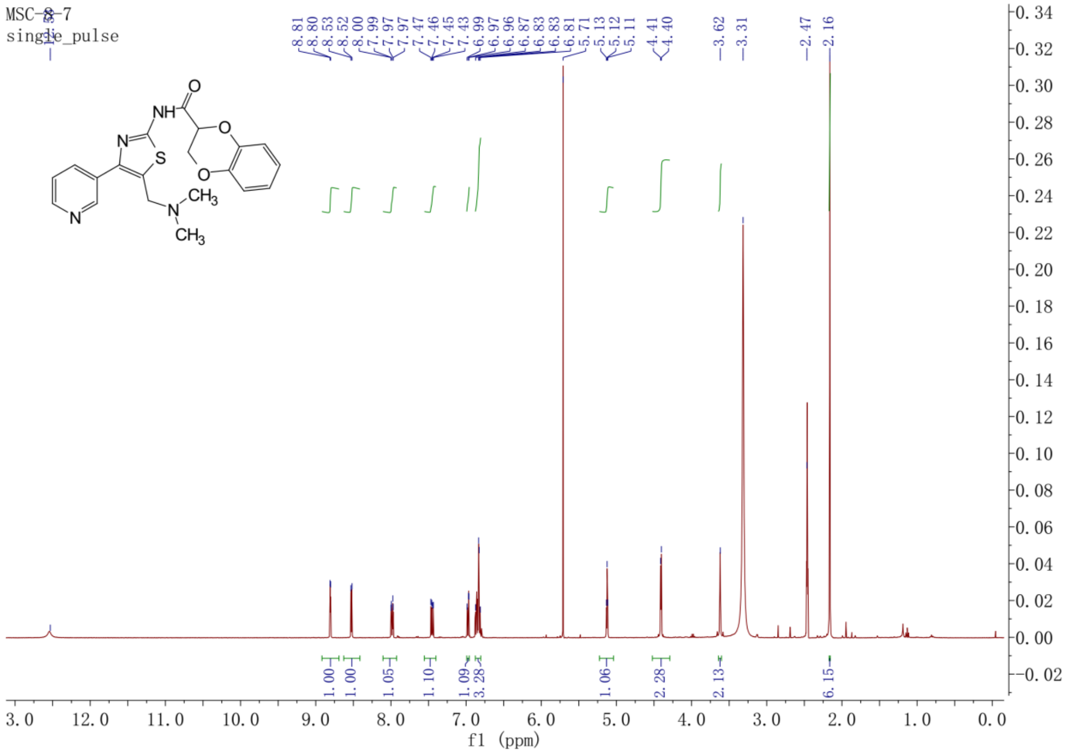

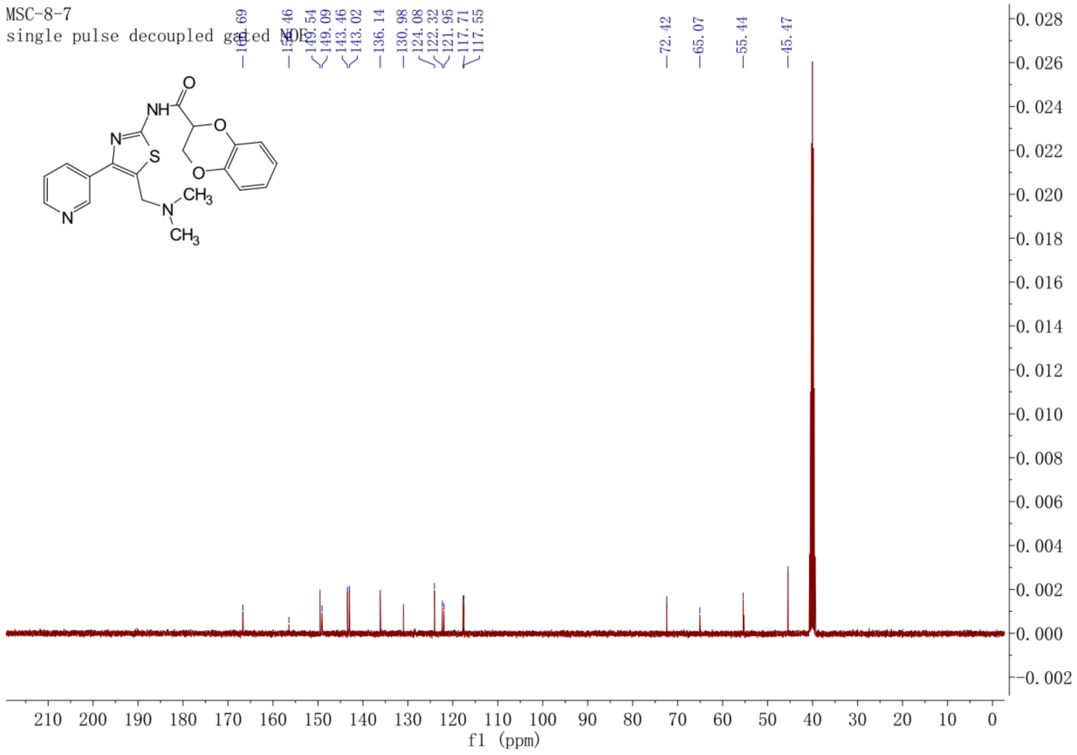

Figure S10. ^{1}H-NMR and ^{13}C-NMR spectrum of **4j**.

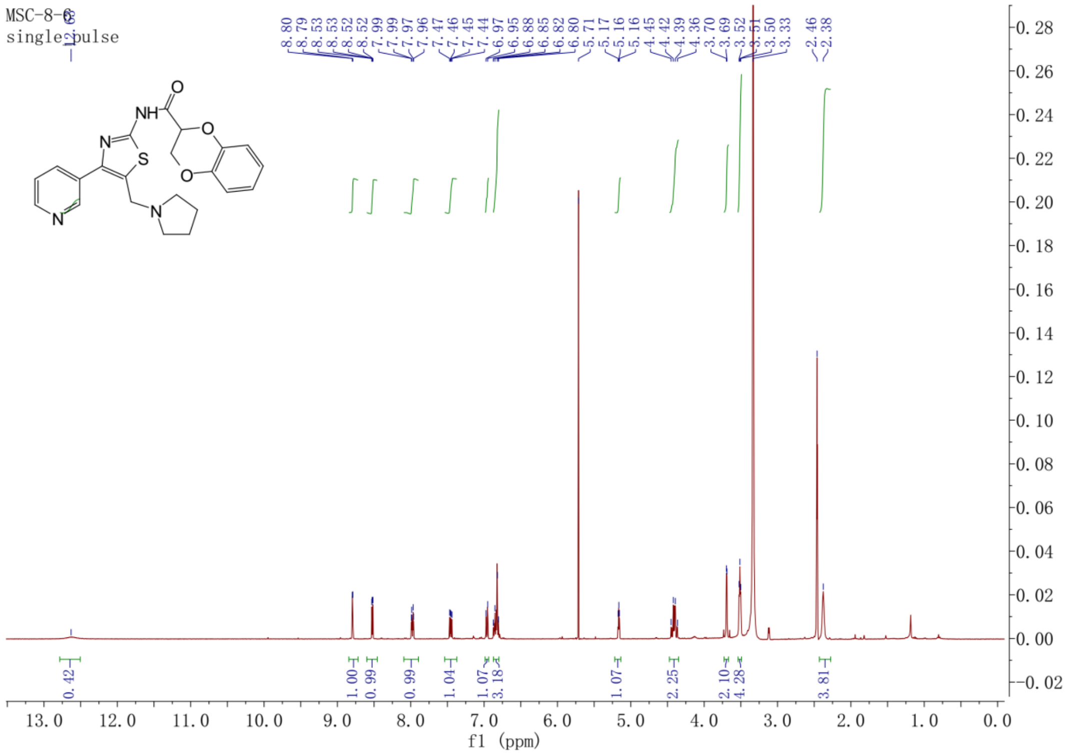

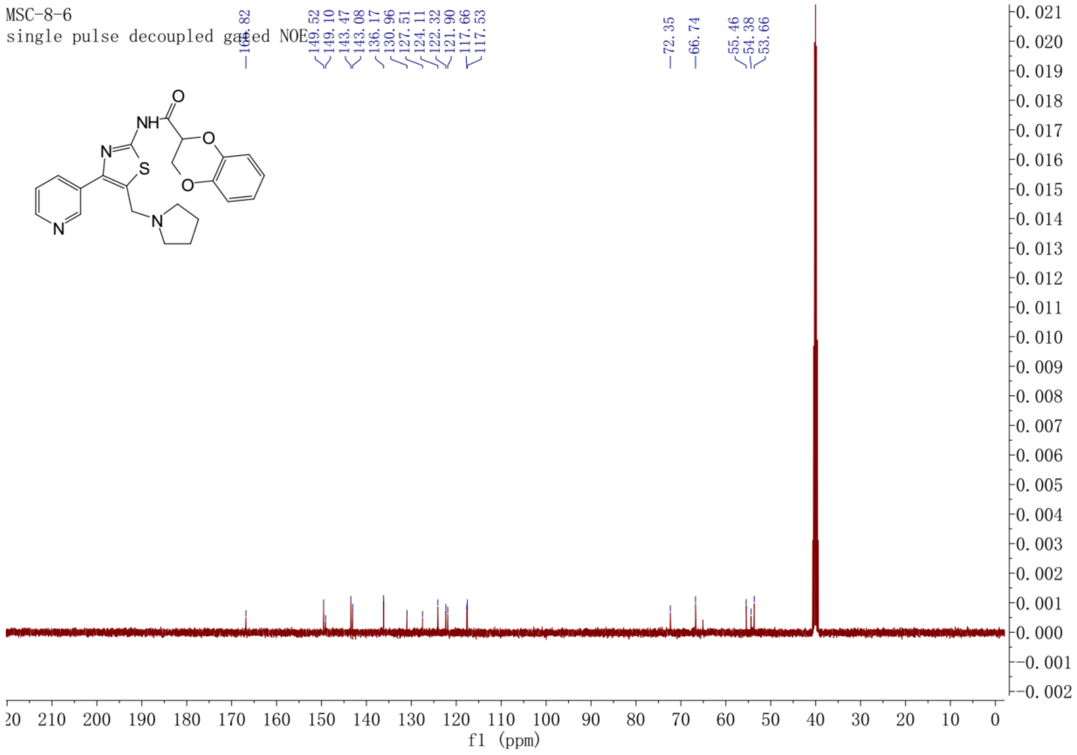

Figure S11. ^{1}H-NMR and ^{13}C-NMR spectrum of **4k**.

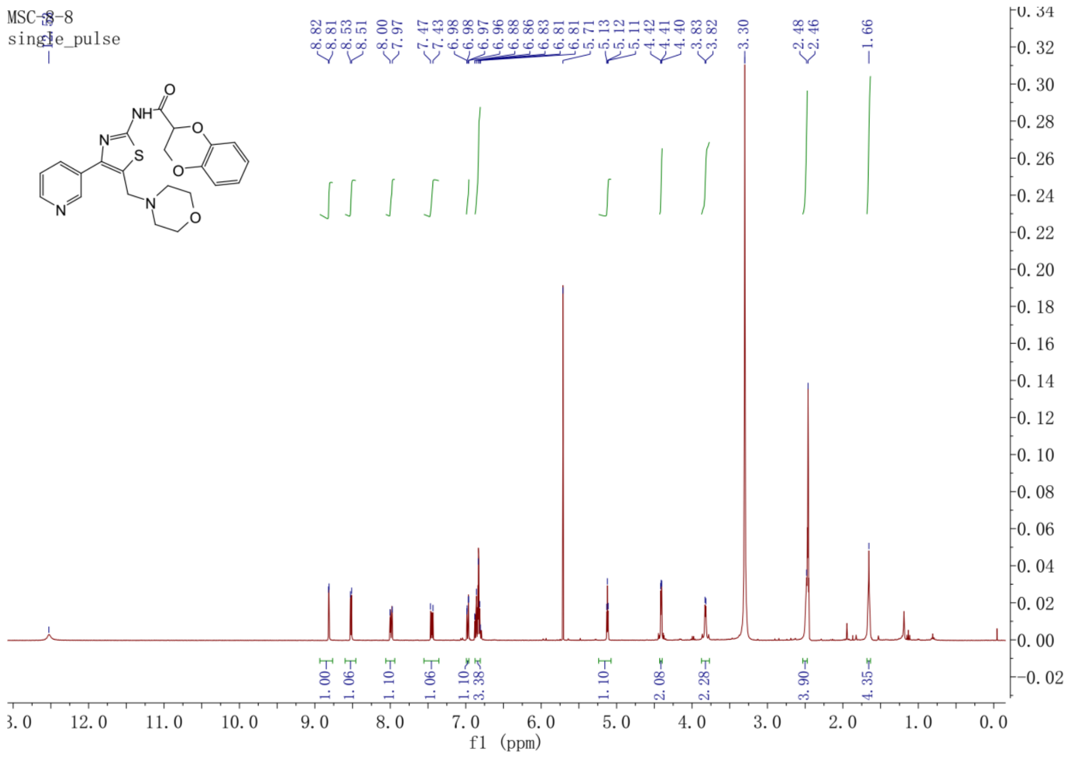

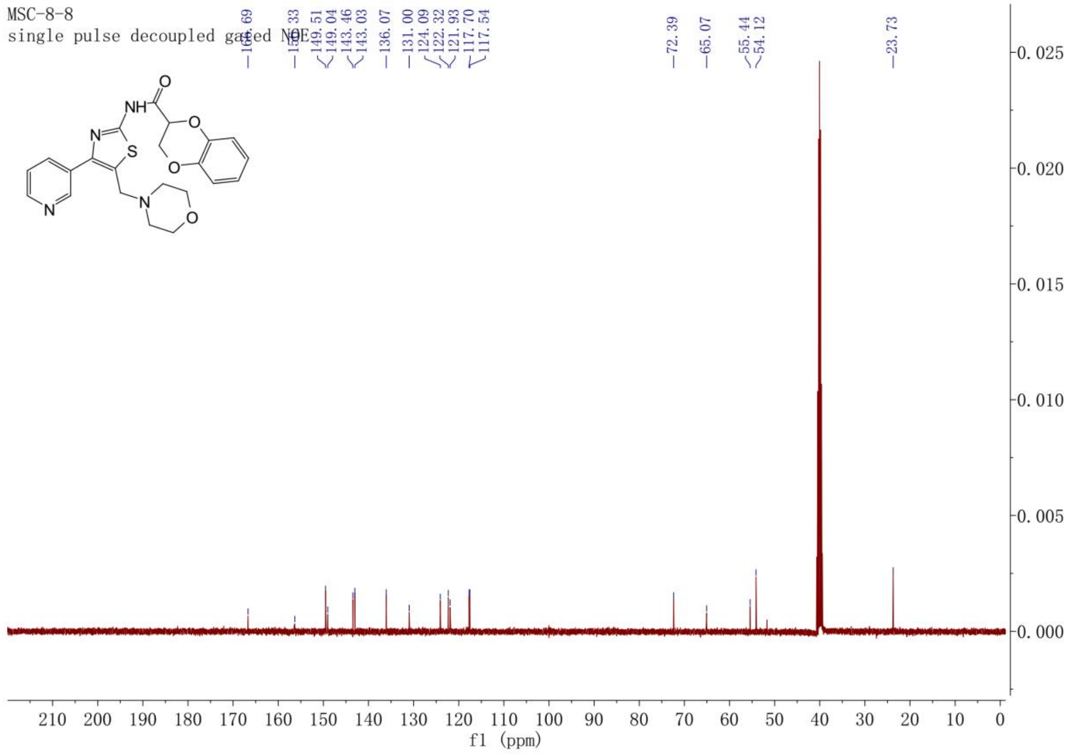

Figure S12. ^{1}H-NMR and ^{13}C-NMR spectrum of **4l**.

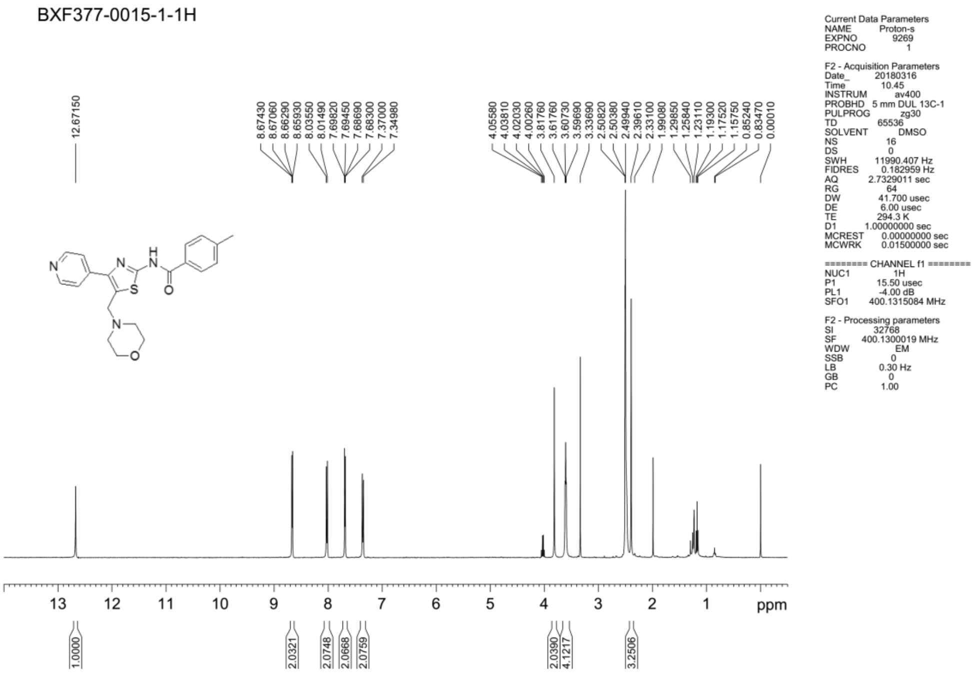

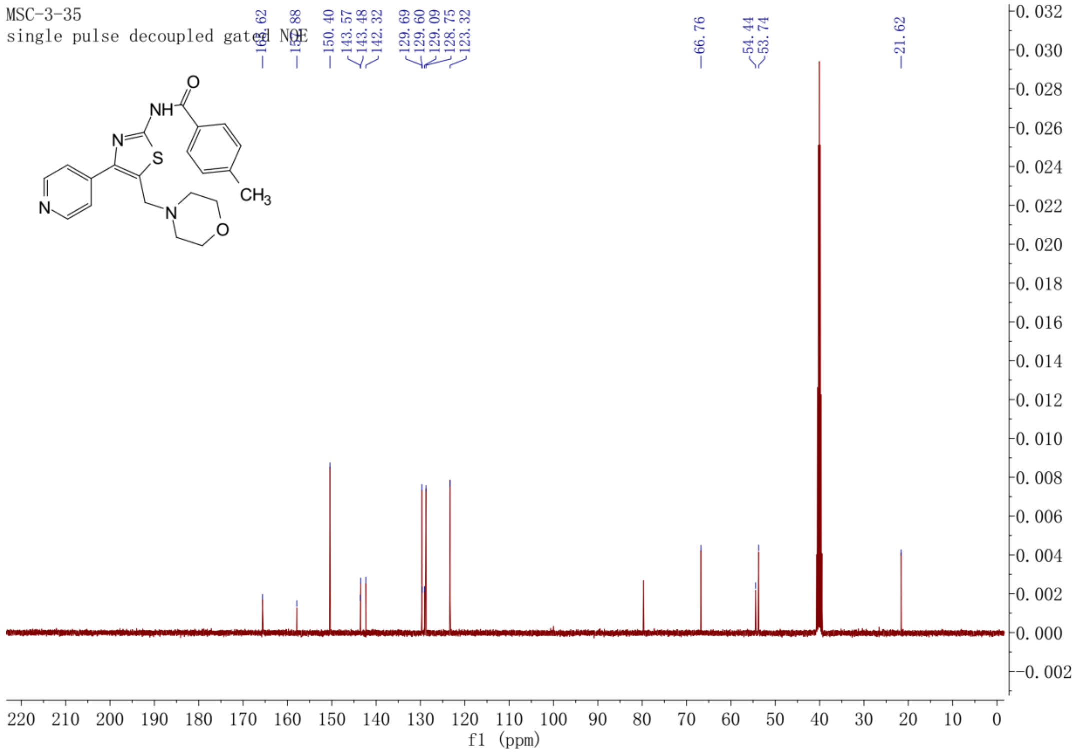

Figure S13. ^{1}H-NMR and ^{13}C-NMR spectrum of **4m.**

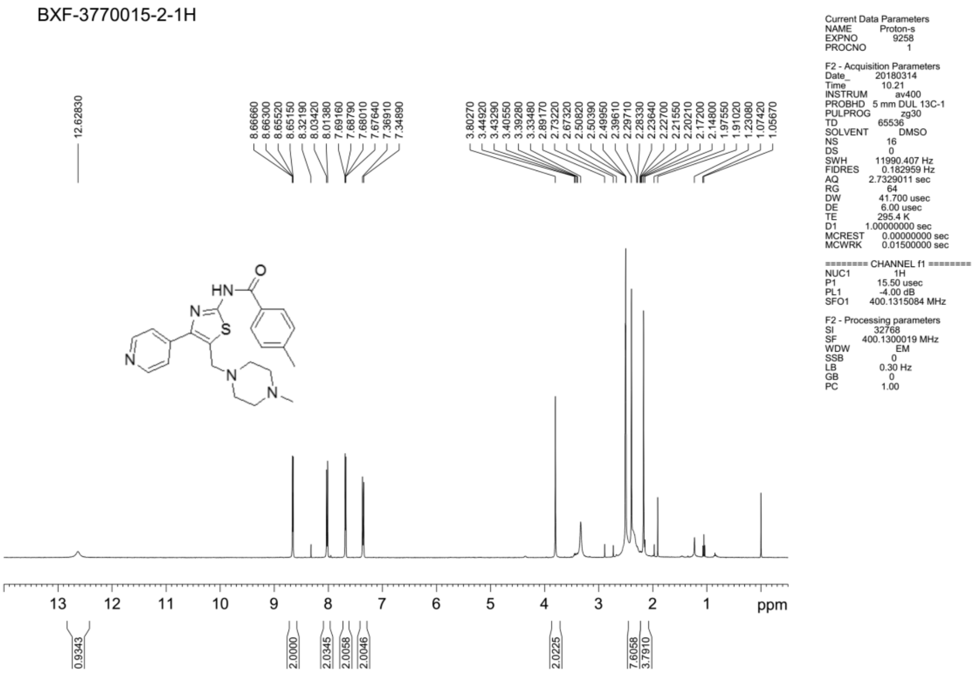

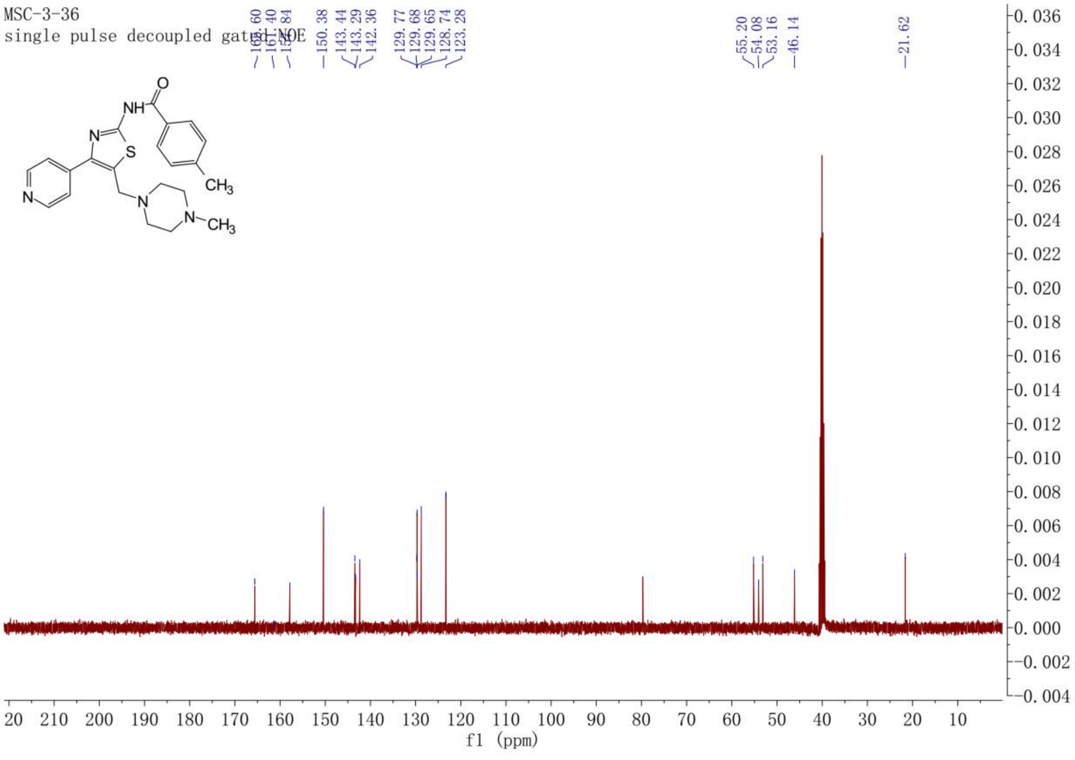

Figure S14. ^{1}H-NMR and ^{13}C-NMR spectrum of **4n**.

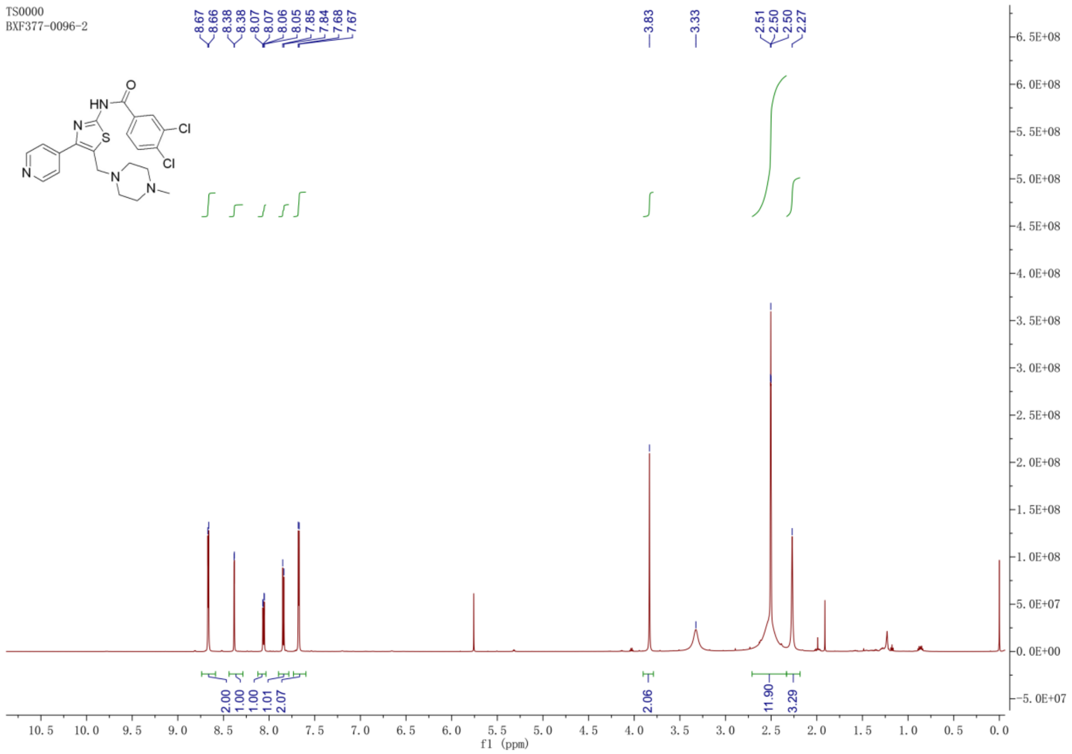

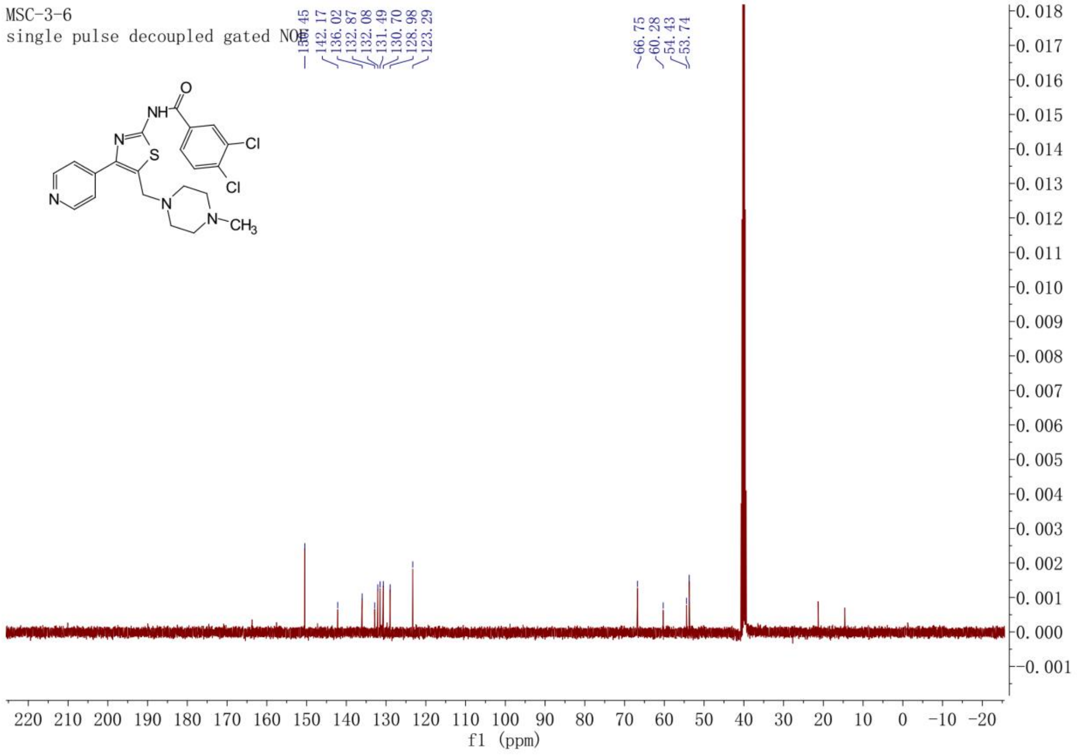

Figure S15. ^{1}H-NMR and ^{13}C-NMR spectrum of **4o**.

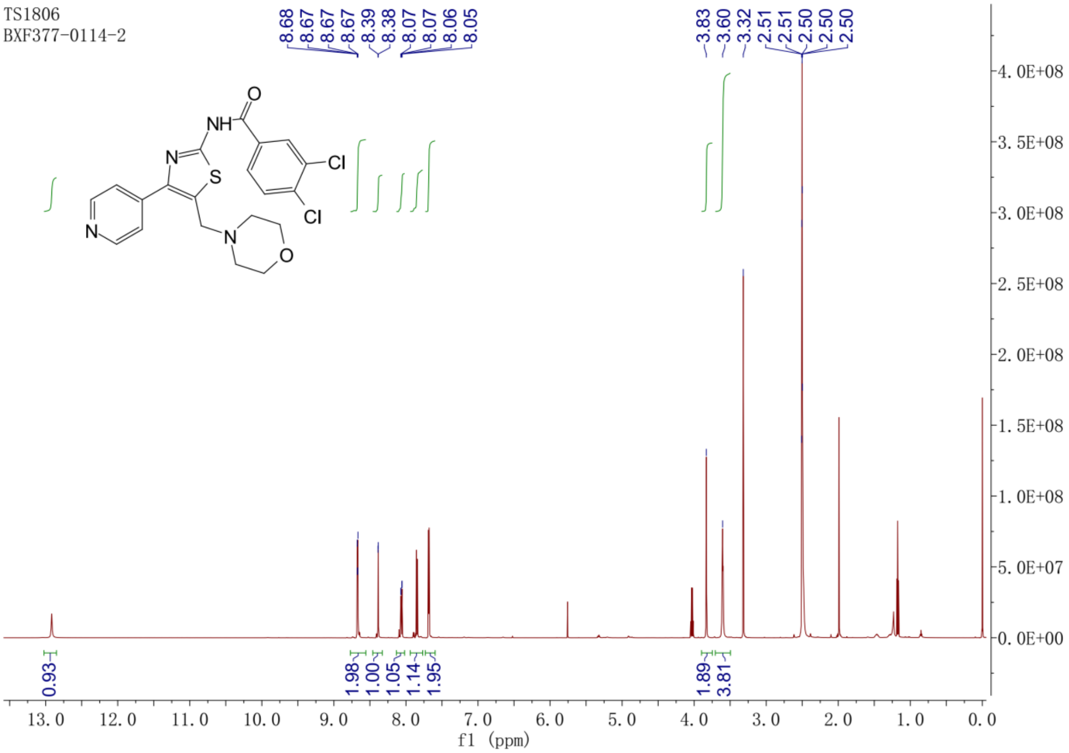

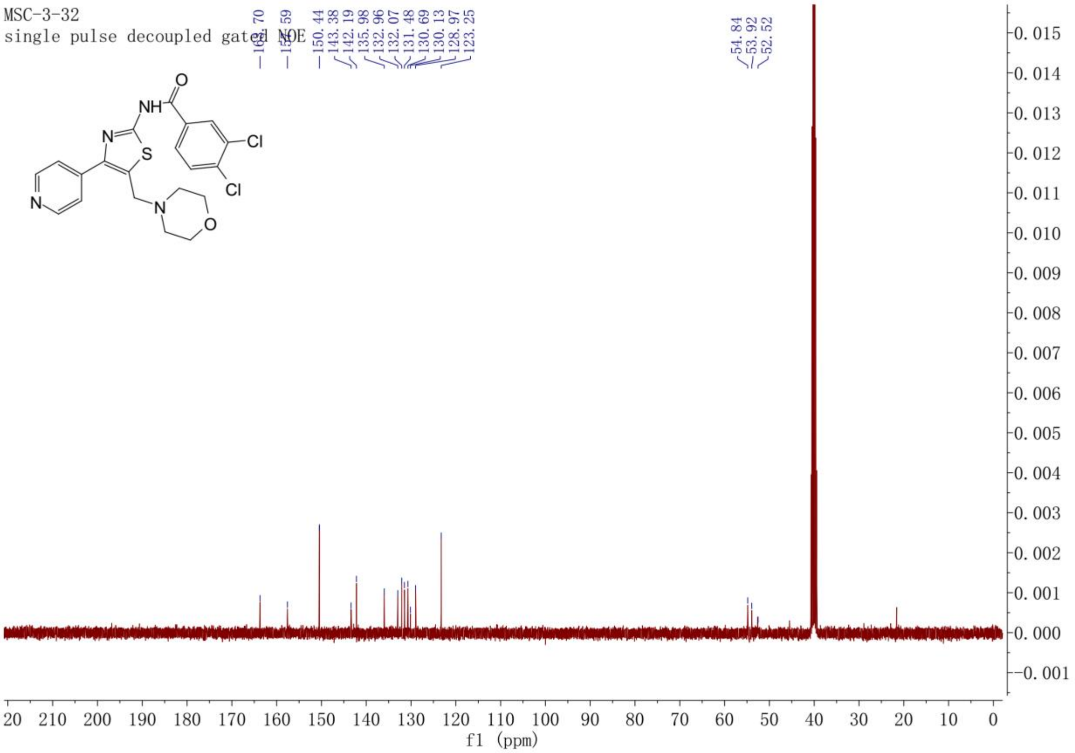

Figure S16. ^{1}H-NMR and ^{13}C-NMR spectrum of **4p**.

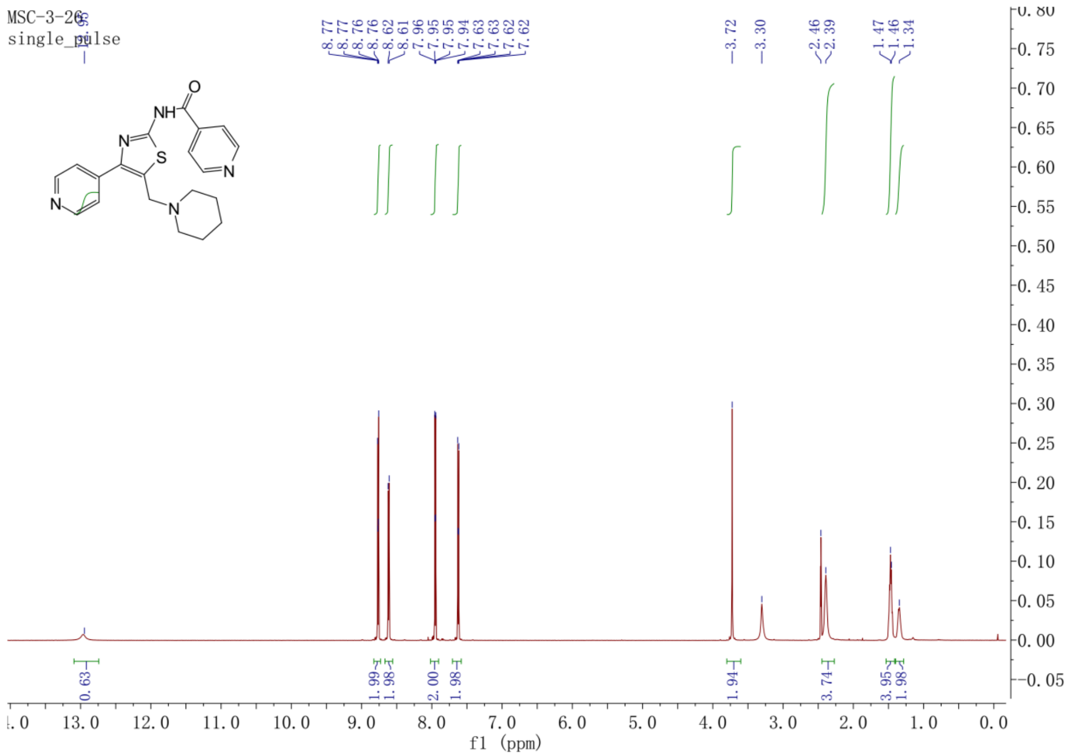

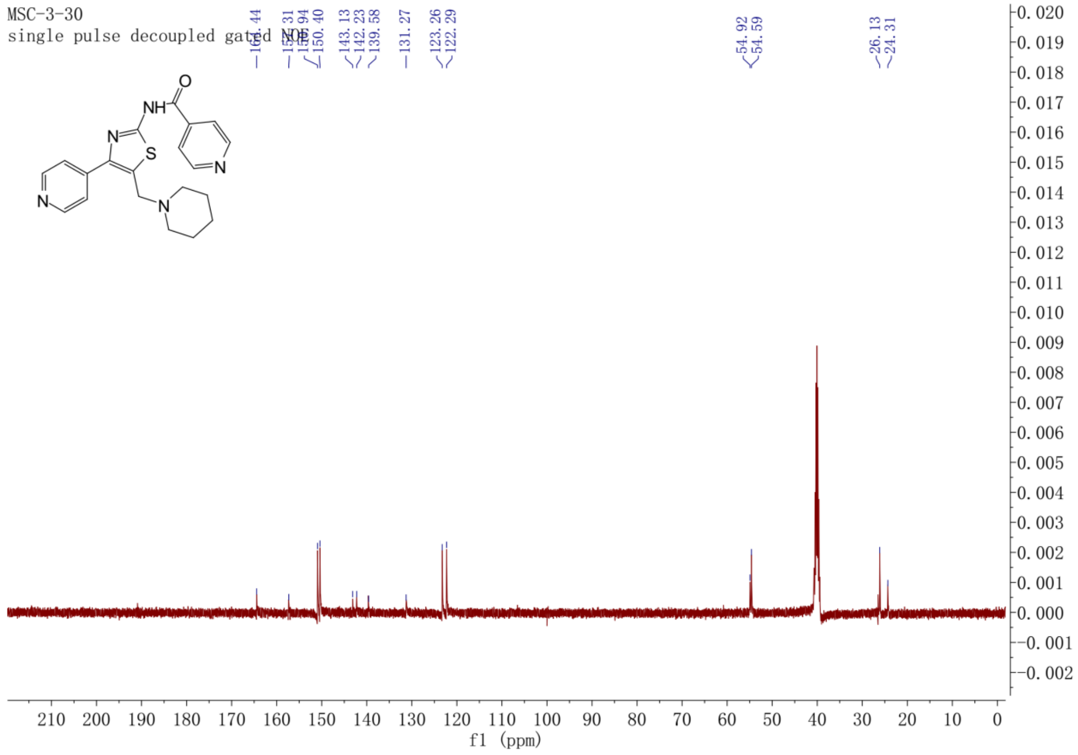

Figure S17. ^{1}H-NMR and ^{13}C-NMR spectrum of **4q**.

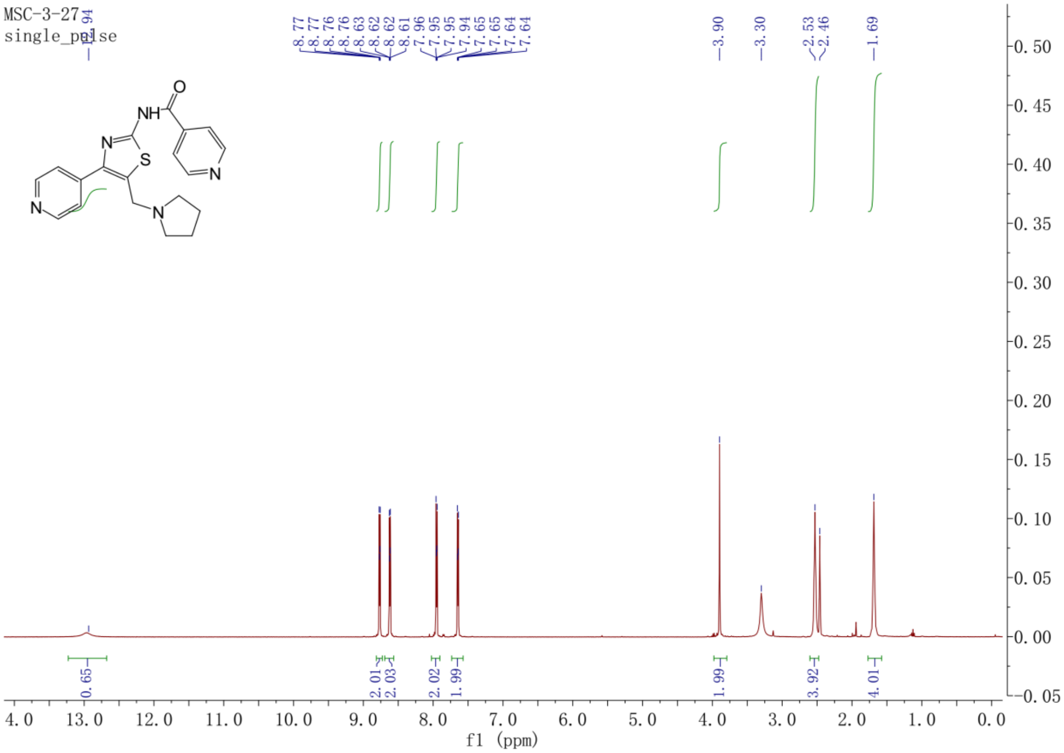

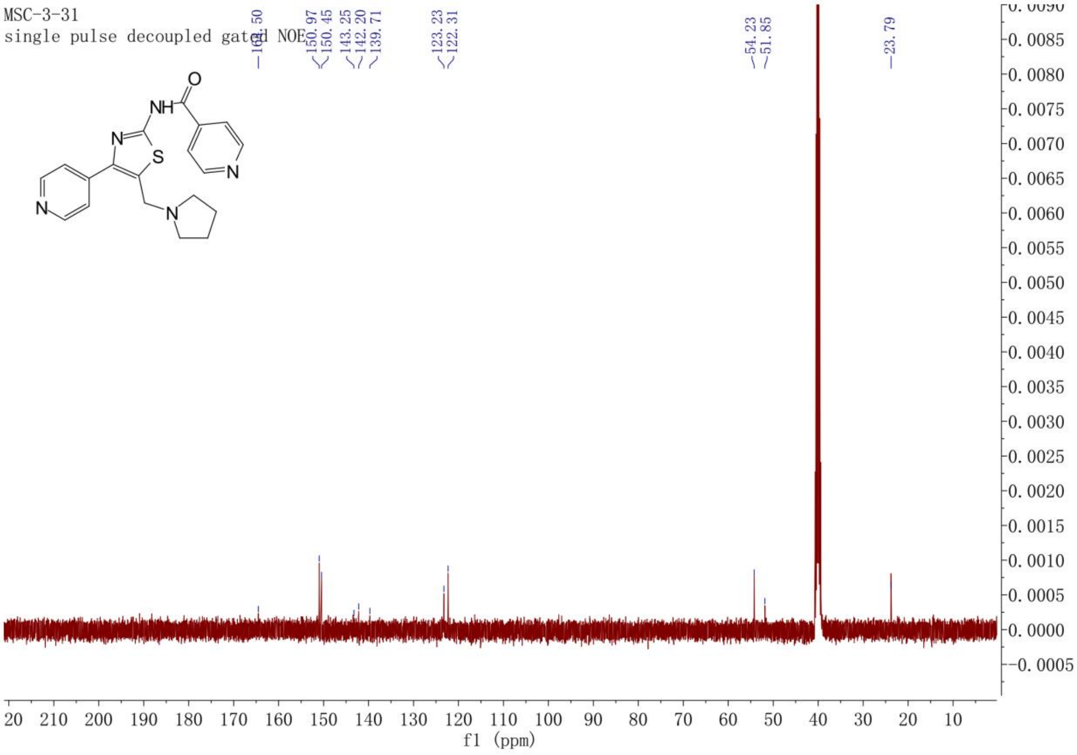

Figure S18. ^{1}H-NMR and ^{13}C-NMR spectrum of **4r**.

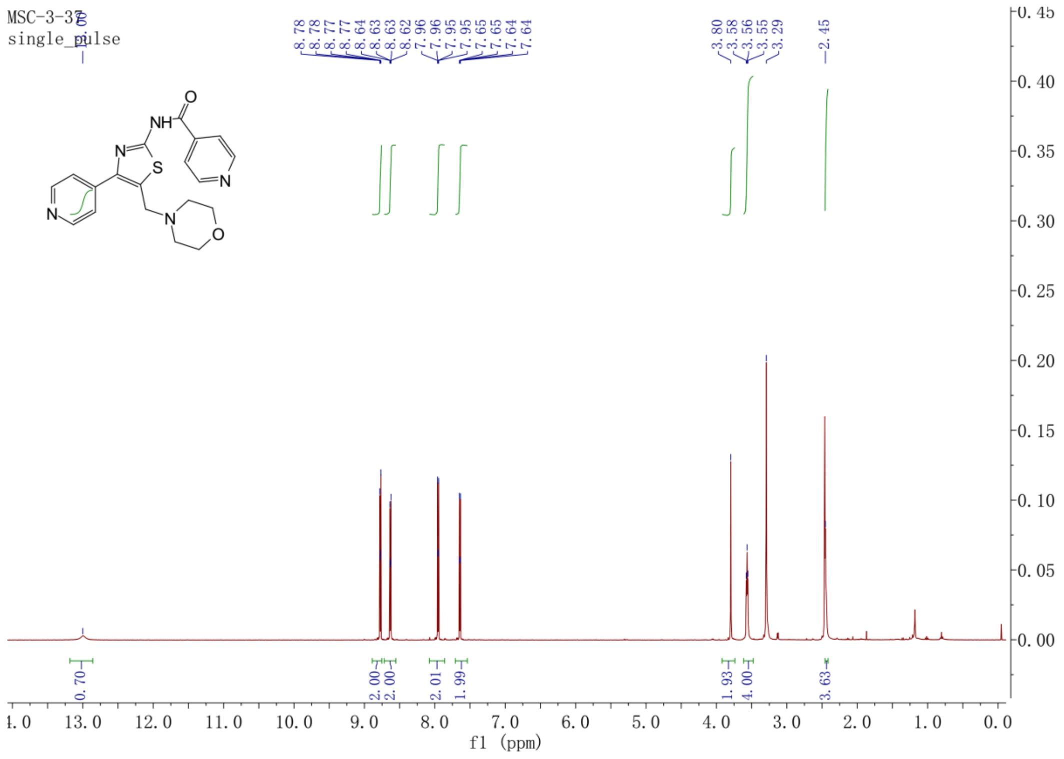

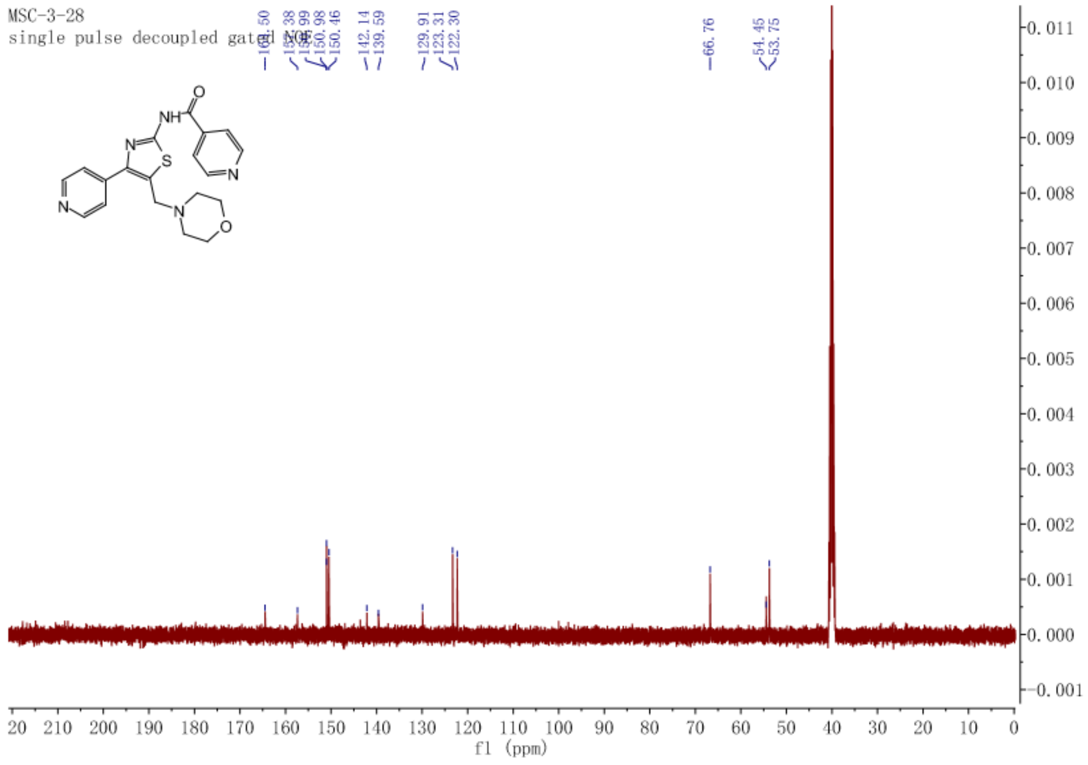

Figure S19. ^{1}H-NMR and ^{13}C-NMR spectrum of **4s**.

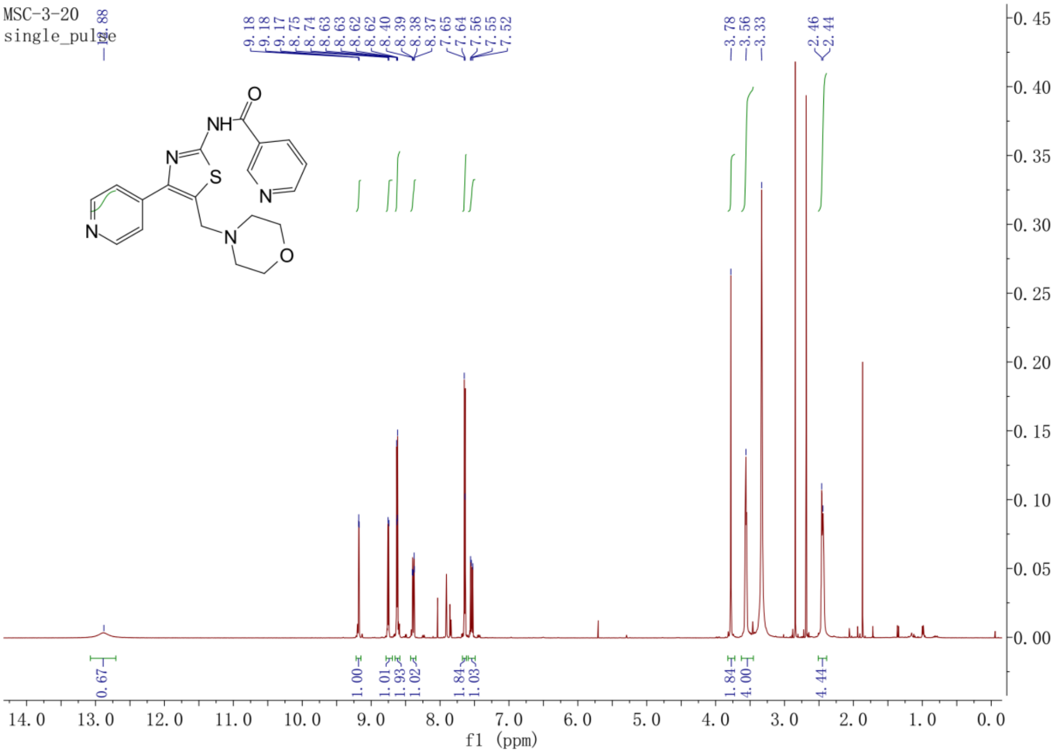

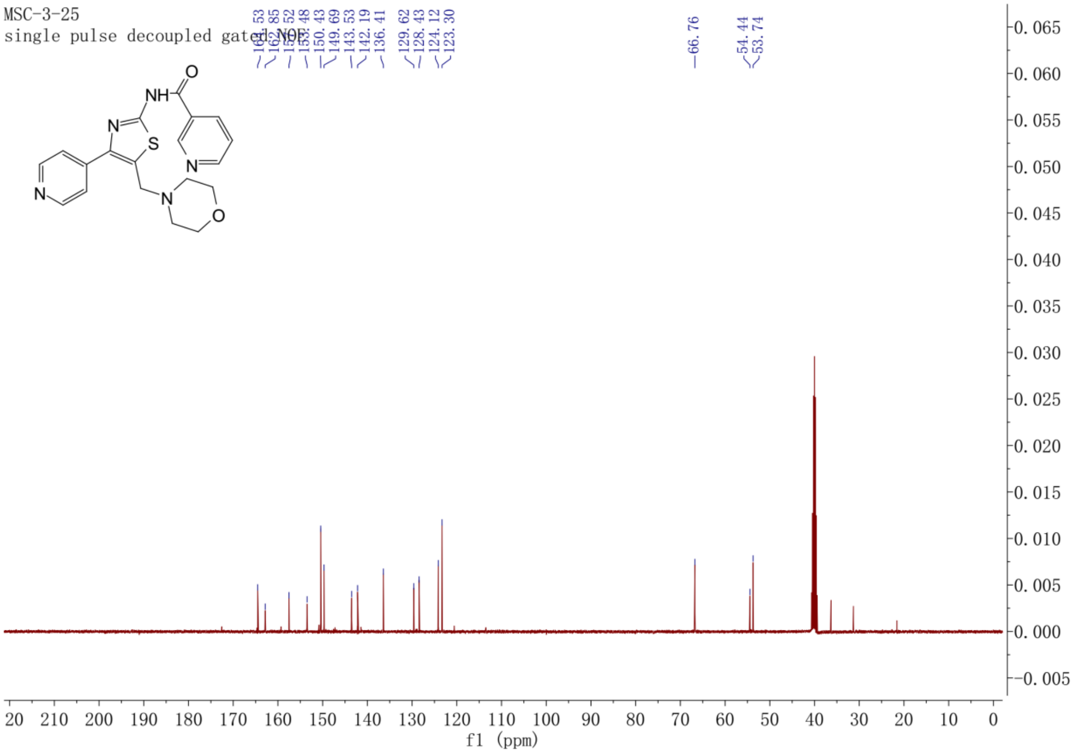

Figure S20. ^{1}H-NMR and ^{13}C-NMR spectrum of **4t**.

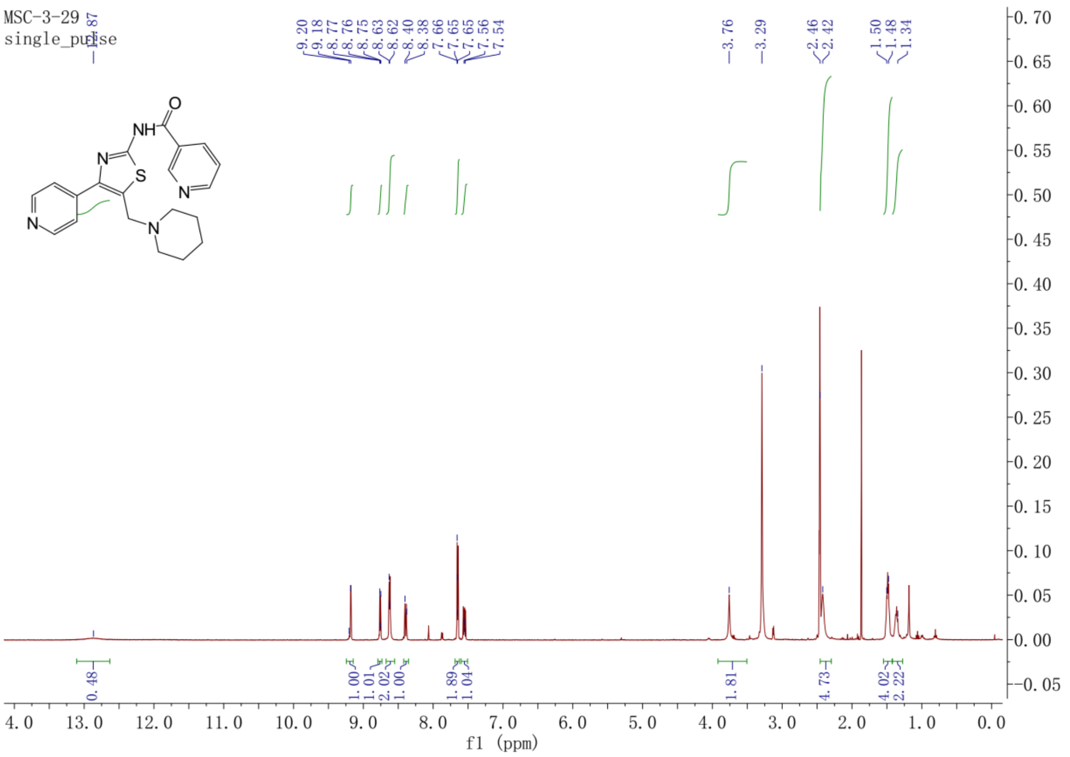

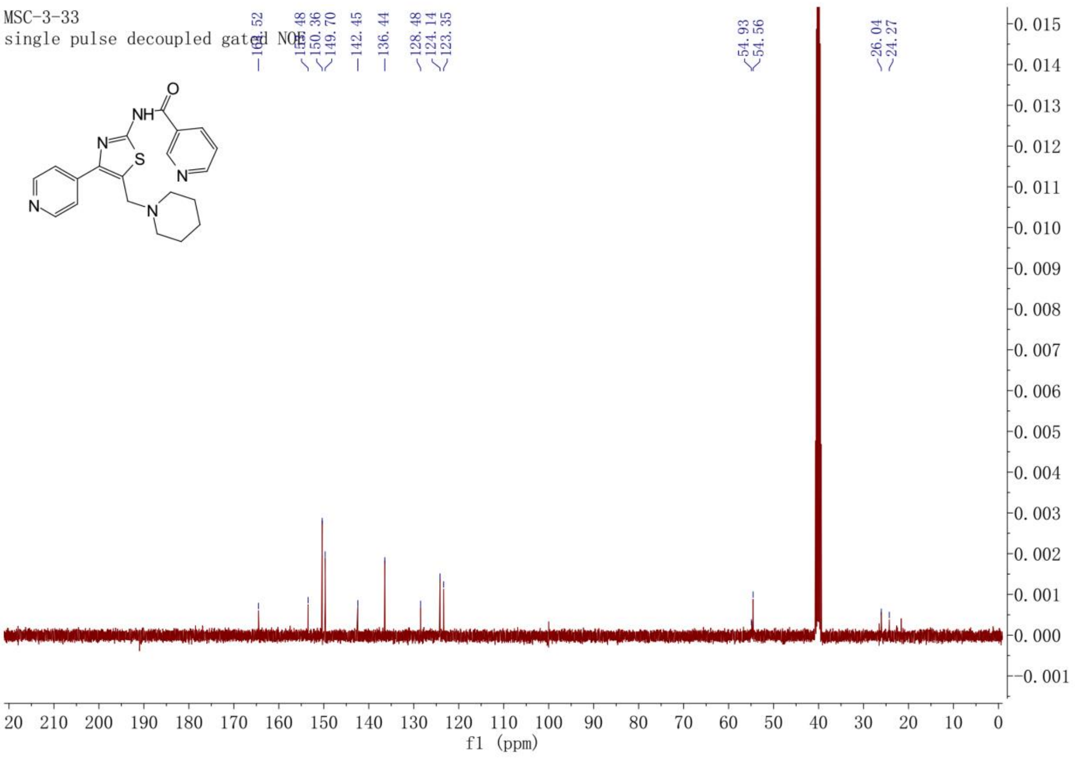

Figure S21. ^{1}H-NMR and ^{13}C-NMR spectrum of **4u**.

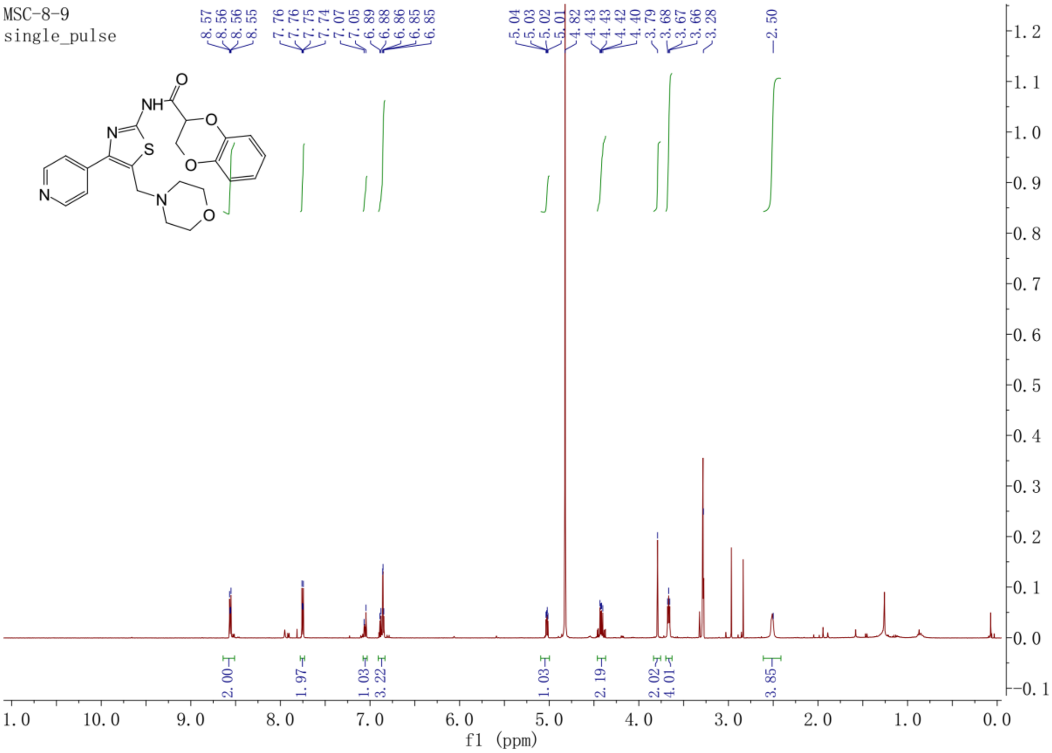

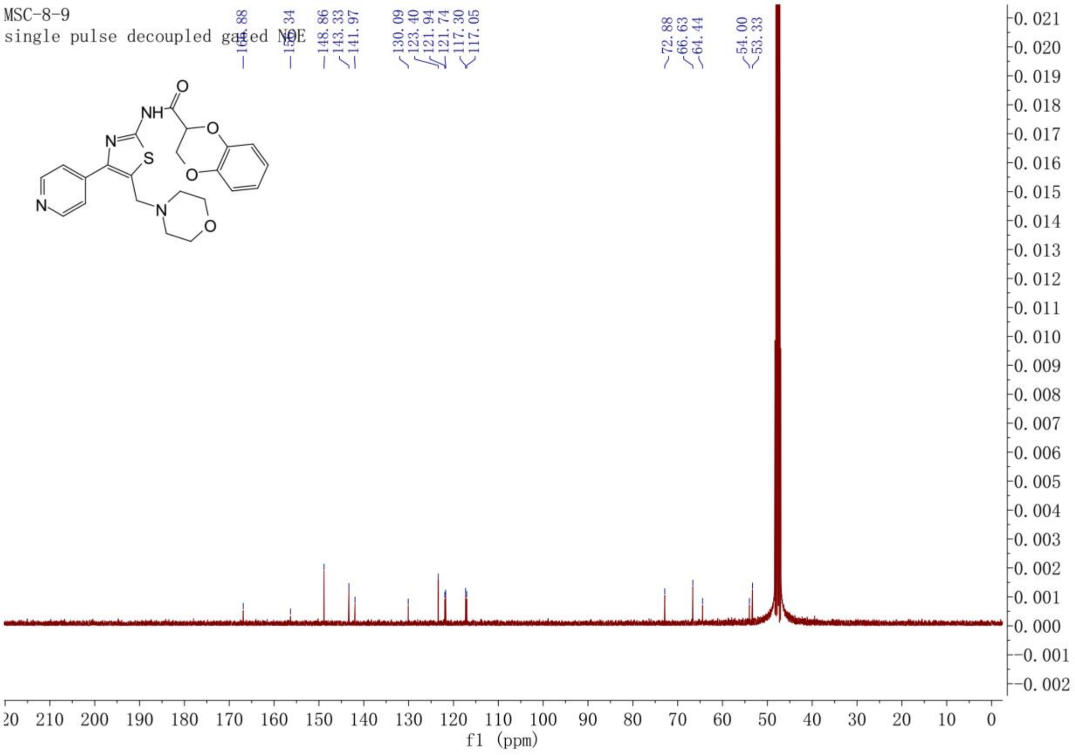

Figure S22. ^{1}H-NMR and ^{13}C-NMR spectrum of **4v**.
